# Cognitive Behavioral Therapy for Insomnia in Pain Management for Nonspecific Chronic Spinal Pain: A Randomized Clinical Trial

**DOI:** 10.1001/jamanetworkopen.2024.25856

**Published:** 2024-08-09

**Authors:** Anneleen Malfliet, Liesbet De Baets, Thomas Bilterys, Eveline Van Looveren, Olivier Mairesse, Barbara Cagnie, Mira Meeus, Maarten Moens, Dorien Goubert, Wouter Munneke, Lieven Daneels, Kelly Ickmans, Steven Kamper, Jo Nijs

**Affiliations:** 1 Pain in Motion Research Group, Department of Physiotherapy, Human Physiology and Anatomy, Faculty of Physical Education & Physiotherapy, Vrije Universiteit Brussels, Brussels, Belgium; 2 Research Foundation–Flanders, Brussels, Belgium; 3 Chronic Pain Rehabilitation, Department of Physical Medicine and Physiotherapy, University Hospital Brussels, Brussels, Belgium; 4 Pain in Motion International Research Consortium; 5 Department of Rehabilitation Sciences and Physiotherapy, Faculty of Medicine and Health Sciences, Ghent University, Ghent, Belgium; 6 Brain, Body and Cognition, Faculty of Psychology and Educational Sciences, Vrije Universiteit Brussel, Brussels, Belgium; 7 Department of Rehabilitation Sciences and Physiotherapy, Faculty of Medicine and Health Sciences, University of Antwerp, Wilrijk, Belgium; 8 Department of Neurosurgery and Radiology, University Hospital Brussels, Brussels, Belgium; 9 Stimulus Research Group, Vrije Universiteit Brussels, Brussels, Belgium; 10 Center of Neurosciences, Vrije Universiteit Brussels, Brussels, Belgium; 11 Department of Sport and Rehabilitation Sciences, University of Liège, Liège, Belgium; 12 Movement & Nutrition for Health & Performance Research Group, Department of Movement and Sport Sciences, Faculty of Physical Education and Physiotherapy, Vrije Universiteit Brussels, Brussels, Belgium; 13 School of Health Sciences, University of Sydney, Camperdown, New South Wales, Australia; 14 Nepean Blue Mountains Local Health District, Sydney, New South Wales, Australia; 15 Department of Health and Rehabilitation, Unit of Physiotherapy, Institute of Neuroscience and Physiology, Sahlgrenska Academy, University of Gothenburg, Gothenburg, Sweden

## Abstract

**Importance:**

Insomnia is highly prevalent in patients with nonspecific chronic spinal pain (nCSP). Given the close interaction between insomnia and pain, targeting sleep problems during therapy could improve treatment outcomes.

**Objective:**

To evaluate the effectiveness of cognitive behavioral therapy for insomnia (CBTi) integrated in best-evidence pain management (BEPM) vs BEPM only in patients with nCSP and insomnia.

**Design, Setting, and Participants:**

A multicenter randomized clinical trial with 1-year follow-up was conducted between April 10, 2018, and April 30, 2022. Data and statistical analysis were performed between May 1, 2022, and April 24, 2023. Patients with nCSP and insomnia were evaluated using self-report and at-home polysomnography, to exclude underlying sleep pathologic factors. Participants were treated at the University Hospital Brussels or University Hospital Ghent, Belgium. Intention-to-treat analysis was performed.

**Interventions:**

Participants were randomized to either CBTi-BEPM or BEPM only. Both groups received 18 treatment sessions over 14 weeks. The CBTi-BEPM treatment included 6 CBTi sessions and 12 BEPM sessions. The BEPM treatment included pain neuroscience education (3 sessions) and exercise therapy (9 sessions in the CBTi-BEPM group, 15 sessions in the BEPM-only group).

**Main Outcomes and Measures:**

The primary outcome was change in mean pain intensity (assessed with Brief Pain Inventory [BPI]) at 12 months after the intervention. Exploratory secondary outcomes included several pain- and sleep-related outcomes. Blinded outcome assessment took place at baseline, posttreatment, and at 3-, 6-, and 12-month follow-up.

**Results:**

A total of 123 patients (mean [SD] age, 40.2 [11.18] years; 84 women [68.3%]) were included in the trial. In 99 participants (80.5%) with 12-month BPI data, the mean pain intensity at 12 months decreased by 1.976 points (reduction of 40%) in the CBTi-BEPM group and 1.006 points (reduction of 24%) points in the BEPM-only group. At 12 months, there was no significant difference in pain intensity change between groups (mean group difference, 0.970 points; 95% CI, −0.051 to 1.992; Cohen *d*, 0.340). Treatment with CBTi-BEPM resulted in a response for BPI average pain with a number needed to treat (NNT) of 4 observed during 12 months. On a preliminary basis, CBTi-BEPM was, consistently over time and analyses, more effective than BEPM only for improving insomnia severity (Cohen *d*, 0.551-1.143; NNT for response ranging from 2 to 4, and NNT for remission ranging from 5 to 12), sleep quality (Cohen *d*, 0.466-0.774), beliefs about sleep (Cohen *d*, 0.679-0.849), depressive symptoms (Cohen *d*, 0.374-0.429), and physical fatigue (Cohen *d*, 0.360-0.481). No serious adverse effects were reported.

**Conclusions and Relevance:**

In this randomized clinical trial, adding CBTi to BEPM did not further improve pain intensity reduction for patients with nCSP and comorbid insomnia more than BEPM alone. Yet, as CBTi-BEPM led to significant and clinically important changes in insomnia severity and sleep quality, CBTi integrated in BEPM should be considered in the treatment of patients with nCSP and comorbid insomnia. Further research can investigate the patient characteristics that moderate the response to CBTi-BEPM in terms of pain-related outcomes, as understanding of these moderators may be of utmost clinical importance.

**Trial Registration:**

Clinical Trials.gov Identifier: NCT03482856

## Key Points

**Question:**

Is cognitive behavioral therapy for insomnia integrated in best-evidence pain management (CBTi-BEPM) more effective than BEPM only for improving pain- and sleep-related outcomes in nonspecific chronic spinal pain (nCSP)?

**Findings:**

In a randomized clinical trial including 123 individuals with nCSP, no statistically significant effect was noted with CBTi-BEPM vs BEPM only on pain intensity. On a preliminary basis, CBTi-BEPM was, consistently over time and analyses, more effective than BEPM only for improving insomnia severity, sleep quality, beliefs about sleep, depressive symptoms, and physical fatigue.

**Meaning:**

The findings of this trial suggest that CBTi integrated in pain management may be considered in the treatment of patients with nCSP and comorbid insomnia.

## Teaser

This randomized clinical trial evaluates the use of cognitive behavioral therapy for insomnia vs best-evidence pain management to manage nonspecific chronic spinal pain in patients with comorbid insomnia.

## Introduction

Nonspecific chronic spinal pain (nCSP) is a large socioeconomic health problem and the leading cause of years lived with disability worldwide.^1^ Nonspecific chronic spinal pain is a multidimensional problem^1^ in which insomnia has a major role.^2^ Insomnia is defined as sleep dissatisfaction with difficulties initiating, maintaining, or returning to sleep for more than 3 days per week for more than 3 months, with a clear influence on daytime functioning.^3^ The prevalence of comorbid clinical insomnia in chronic pain conditions varies between 53% and 90%.^4,5,6,7,8^ Specifically in nCSP, insomnia rates exceed 50%,^9,10^ resulting in detrimental daytime effects, such as decline in memory and decreased quality of life (QOL).^11^

Because of the complex bidirectional sleep-pain association,^12,13^ the presence of insomnia may impede treatment effects in nCSP.^14,15^ Hence, specifically targeting sleep in these patients by using cognitive behavioral therapy for insomnia (CBTi) integrated in pain management may increase treatment effectiveness. Cognitive behavioral therapy for insomnia is a nonpharmacologic, multicomponent intervention aiming at changing unhelpful sleep-related attitudes, beliefs, and behaviors.^16^ International guidelines recommend CBTi as first-line treatment for insomnia based on its well-established positive effects on sleep outcomes,^17,18^ such as sleep quality, insomnia severity, and fatigue, in individuals with chronic pain.^19,20^ However, less favorable effects of CBTi as a stand-alone treatment for pain are reported in patients with nCSP,^21,22^ which may be explained by the absence of integrating CBTi in best-evidence pain management (CBTi-BEPM).

To our knowledge, this is the first fully powered randomized clinical trial evaluating the effectiveness of CBTi-BEPM vs BEPM only for reducing pain intensity up to 12 months after intervention in patients with nCSP and insomnia. Additionally, on an exploratory basis, this study examines whether CBTi-BEPM vs BEPM only can improve other pain-related and sleep-related outcomes, physical activity, depressive symptoms, anxiety, and QOL.

## Methods

### Design and Blinding

This triple-blind study (participants, assessors, and statistician) was approved by the ethics committee at the University Hospital of Ghent and University Hospital of Brussels. A multicenter randomized clinical trial with 1-year follow-up was conducted between April 10, 2018, and April 30, 2022. Data and statistical analysis were performed between May 1, 2022, and April 24, 2023. A detailed study protocol can be found in Supplement 1 and elsewhere.^23^ Blinding of assessors and participants was evaluated. This study followed the Consolidated Standards of Reporting Trials (CONSORT) reporting guideline.

### Study Population

Patients with nCSP and insomnia aged 18 to 65 years were recruited via the participating universities, university hospitals, primary care practices, occupational health services, public places, advertisements, and social media. Patients with nCSP and insomnia were evaluated using self-report and at-home polysomnography, to exclude underlying sleep pathologic factors. Participants were treated at the University Hospital Brussels or University Hospital Ghent, Belgium. Details on eligibility criteria and the polysomnography (PSG) assessment used for identifying insomnia and excluding severe underlying sleep pathologic factors are included in eTable 1 and the eMethods in Supplement 2 and elsewhere.^23^

### Randomization

Randomization was computer-generated at the Ghent University Biostatistics Unit by an independent investigator. Block randomization (1:1) was used for the 2 treatment centers (University Hospital of Ghent and University Hospital of Brussels) separately, with stratification for sex (male and female) and dominant pain location (neck and lower back).^13^ Paper strips indicating group assignment were placed in sequentially numbered, opaque, sealed envelopes. An independent researcher, the only one with access to the envelopes, wrote the participant’s initials on the envelope before opening it, ensuring concealed randomization.

### Outcome Measures

Outcome assessment was performed at baseline, immediately after treatment, and at 3-, 6-, and 12-month follow-up. The primary end point was 12-month follow-up. For details, see the a priori published protocol^23^ and in Supplement 1.

The primary clinical outcome was mean pain intensity, assessed using the Brief Pain Inventory (BPI)^24^ item mean pain intensity in the last 24 hours evaluated on an 11-point numeric rating scale (minimal clinically important difference [MCID] = 30% decrease).^15,25^

Exploratory secondary pain-related outcomes comprised self-reported pain outcomes, including BPI worst and least pain intensity (past 24 hours), BPI pain intensity now (ie, at the time of assessment), BPI pain severity and interference, and symptoms of central sensitization, assessed via the Central Sensitization Inventory (>40 of 100 indicates the presence of central sensitization-related symptoms).^26,27^ Pressure pain thresholds (MCID = increase >15%^28^) were assessed with a digital pressure algometer (Wagner Instruments) randomly applied at the painful location and 2 remote locations.^29,30^

Exploratory secondary sleep-related outcomes included data on perceived sleep quality assessed by the Pittsburgh Sleep Quality Index (cutoff = 6 of 21 points; MCID = 3 points^31,32,33^); insomnia severity assessed by the Insomnia Severity Index (cutoff = 14 of 28 points; ie, scores ≤14 considered as remittance; MCID = 6 points; ie, reduction ≥6 points qualifies as response^32,34,35^); sleep- and insomnia-related cognition, assessed by the Dysfunctional Beliefs and Attitudes About Sleep questionnaire^36,37^; sleep propensity measured by the Epworth Sleepiness Scale^38^; and mental and physical fatigue assessed by the Brugmann Fatigue Scale.^39^ Additionally, objective sleep outcomes were assessed using at-home PSG (portable Alice PDX, Philips Respironics Inc) and included sleep-onset latency, wake duration after sleep onset, early-morning awakening, time in bed, total sleep time, sleep efficiency, percentage in rapid eye movement (REM) and non-REM sleep, and number of arousals (eMethods in Supplement 2  provides details).

Other explorative secondary outcomes included depressive symptoms and anxiety (measured with the Hospital Anxiety and Depression Rating Scale, cutoff = 7 of 21 points; MCID = 1.7 points^40,41,42^); objective physical activity–related outcomes (recorded over 7 consecutive days using 3-axis accelerometers, GT9X-BT, Actigraph^43,44^), including step count and percentage of time in sedentary, light, moderate, and moderate/vigorous physical activity (analyzed using ActiLife6, Actigraph Corporation LLC); health-related QOL (ie, the 36-item Short-Form Health Survey^45^); adverse events, classified as serious (led to death, life-threatening, required hospitalization, prolonged hospitalization, and led to prolonged or major disability).

### Intervention

Both groups received 18 sessions of approximately 30 minutes of therapy during 14 weeks. All sessions were delivered by physical therapists (all with Master of Science degree), and were one-on-one, individualized sessions (except for 1 group session of 1 hour) using principles of person-centered care and applying guidance toward self-management (eTable 2 in Supplement 2).

As the experimental intervention, CBTi-BEPM comprised 6 sessions of CBTi combined with 12 sessions of BEPM.^46^ The control intervention, BEPM only, comprised 18 sessions of BEPM. The treatment contrast lay in the 6 CBTi sessions, which included sleep education, self-monitoring of sleep patterns, time-in-bed restriction, stimulus control, sleep hygiene, cognitive restructuring, and relaxation.^47^ Details are available in the protocol (Supplement 1 and published work.^46,47,48^ Best-evidence pain management included pain neuroscience education (3 sessions) and cognition-targeted exercise therapy. In CBTi-BEPM, 9 sessions of cognition-targeted exercise therapy were administered, compared with 15 sessions in the BEPM-only intervention, ensuring equivalent therapy and therapist exposure times across treatment arms. Full details are available in the protocol (Supplement 1) and published work.^49^

### Statistical Analysis

Sample size (N = 120) was calculated using G*Power, version 3.1.9.2, based on the effects on pain in a pilot study (effect size *f* = 0.25, α = .05, power = 0.80),^22^ accounting for *F* tests and 20% loss-to-follow-up at 12 months.^50^

All analyses (intention-to-treat) were performed in SPSS, version 24.0 (SPSS Institute Inc). For all outcomes, the change between baseline and other time points was calculated (eg, change 1 = baseline [T0] to posttreatment [T1]; change 2 = T0 to 3-month follow-up [T2]). Differences in the change in mean pain intensity at the 12-month follow-up (change 4, primary outcome at primary end point) and at the other time points were analyzed using a random-intercept fixed-slope linear mixed model (including least significant difference post hoc analyses), with an unstructured covariance matrix. Linear mixed models are a likelihood-based estimation procedure, whereby likely values for missing data are estimated from information contained in the observed data, resulting in nonbiased estimates, providing data are missing at random. The model included treatment, time, and treatment × time as fixed effects together with a random intercept for each patient. Model assumptions were evaluated visually using residual plots. Mean group differences (MGD) with 95% CIs at the different time points, their *P* values, and effect sizes for the intervention comparisons are reported. Level of significance was set at α = .05. Effect sizes were calculated as Cohen *d* (interpreted as >1.3 = very large, 0.80-1.29 = large, 0.50-0.79 = medium, 0.20-0.49 = small, and <0.20 = negligible). The same analysis was used to evaluate the explorative secondary outcomes at the different time points. Significance was determined using 2-sided, unpaired testing.

Different sensitivity analyses were performed. The first sensitivity analyses entailed the assessment of between-group differences using the same analysis, while including baseline levels of mean pain intensity for all pain-related outcomes and baseline level of insomnia severity for sleep-related outcomes as a confounder. The second sensitivity analysis compared the dropout group, including loss-to-follow-up, with the no dropout group by categorizing the entire cohort into 2 subsets, distinguished by their adherence or discontinuation from the trial. A *t* test or its nonparametric equivalent was used to compare the baseline characteristics and data between the 2 groups.

## Results

### Flow of Participants Throughout the Study

A total of 123 people (mean [SD] age, 40.2 [11.18] years; 84 [68.3%] women; 39 [31.7%] men) were randomized (n = 61 experimental; n = 62 control). Full details on the participants’ flow through the study are presented in the Figure. Apart from 13 individuals who dropped out (10.6%; 6 experimental and 7 control), all included participants finalized 18 treatment sessions. Loss-to-follow-up occurred in 7 experimental participants and 2 control participants (7.3%). Details on the data missing per group, outcome, and time point are provided in eTable 3 in Supplement 2. Missing data were mainly attributed to COVID-19 and technical issues. There were no study protocol deviations.^23^ Table 1 and Table 2 present participants’ other characteristics. For success of blinding, see eTable 7 in Supplement 2.

**Figure.**
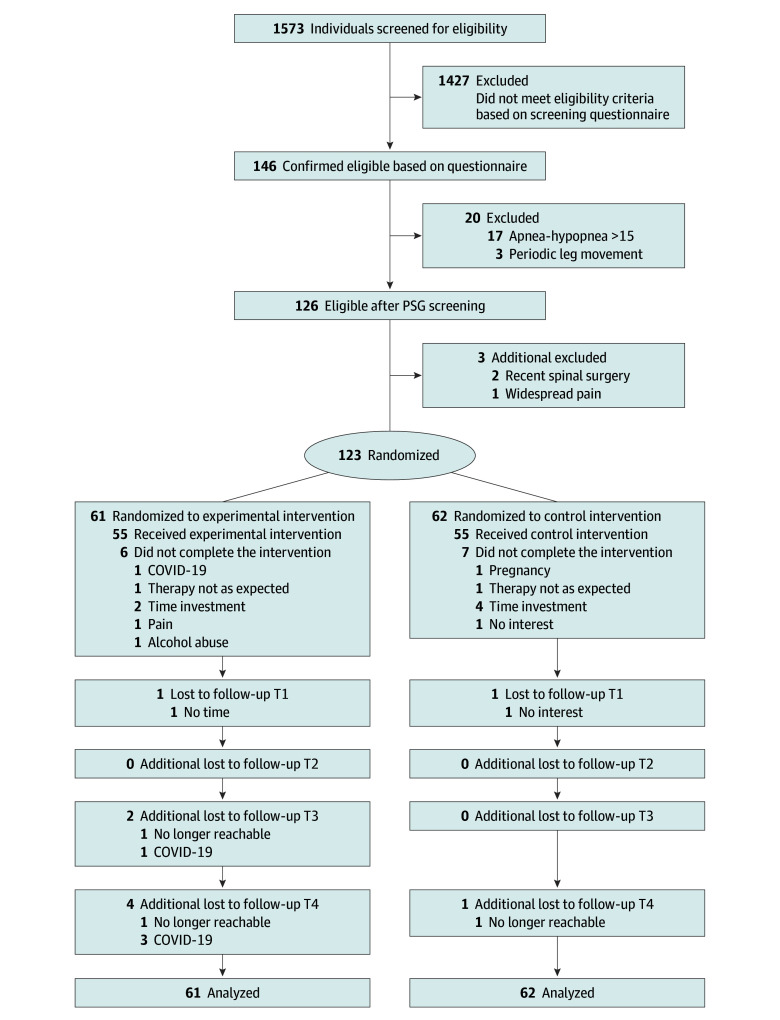
Study Flowchart A total of 123 individuals were included in the analysis because of the use of linear mixed models analysis, which a likelihood-based estimation procedure whereby likely values for missing data are estimated from information contained in the observed data, resulting in nonbiased estimates providing that data are missing at random. PSG indicates polysomnography.

**Table 1.** Baseline Participant Characteristics

Variable	BEPM only (n = 62)			BEPM with CBTi (n = 61)		
	Mean (SD)	Range	IQR	Mean (SD)	Range	IQR
**Demographic characteristics**						
Age, y	39.69 (11.05)	21-61	30.75-49.00	41.03 (11.12)	21-61	32.00-49.50
BMI	23.07 (3.18)	17.04-30.49	24.00-120.00	23.59 (3.10)	15.97-30.11	21.48-25.89
**Pain related**						
Pain duration, mo	82.77 (89.03)	3-444	24.00-120.00	96.72 (102.41)	3-540	24.00-120.00
Brief Pain Inventory^a^						
Mean pain intensity	4.21 (1.94)	1-9	3.00-6.00	5.03 (2.07)	1-9	4.00-7.00
Least pain intensity	1.95 (1.59)	0-9	1.00-2.25	2.44 (1.67)	0-7	1.00-4.00
Worst pain intensity	6.92 (1.65)	1-9	6.00-8.00	7.20 (1.54)	4-10	6.00-8.00
Pain intensity now	3.25 (2.17)	0-8	1.75-4.00	4.31 (2.06)	0-9	3.00-6.00
Pain severity	4.09 (1.47)	0.50-7.00	3.00-5.06	4.75 (1.53)	1.25-8.25	3.75-5.75
Pain interference	2.74 (1.69)	0-7.43	1.43-4.00	3.59 (1.91)	0-7.71	2.07-4.93
Pressure pain threshold, kgf						
Primary (corrected for dominant pain location)	4.87 (2.77)	NA	NA	4.82 (2.23)	NA	NA
Secondary, hand	4.05 (1.57)	1.73-11.24	2.96-4.98	4.17 (1.61)	1.67-9.07	2.94-5.13
Secondary, leg	4.72 (1.86)	1.44-10.29	3.48-5.61	5.20 (1.88)	1.83-10.32	3.84-6.16
Central sensitization inventory^b^ (range, 0-100)	43.21 (11.14)	16.0-70.0	36.00-50.00	44.25 (10.57)	20.0-65.0	36.50-52.00
**Sleep-related**						
Brugmann Fatigue Scale^c^						
Mental component	3.27 (2.49)	0-10	1.00-5.00	3.18 (2.53)	0-10	1.00-5.00
Physical component	3.16 (2.20)	0-9	1.00-5.00	3.05 (2.12)	0-9	2.00-5.00
DBAS^d^	3.00 (0.59)	1.06-4.55	2.69-3.38	3.00 (0.59)	1.69-4.31	2.59-3.38
Epworth Sleepiness Scale^e^	8.24 (4.63)	0-22	5.00-11.00	8.23 (4.70)	1-20	5.00-11.50
Insomnia Severity Index^f^	14.19 (4.09)	4-24	11.75-17.00	16.10 (3.99)	8-27	13.00-19.00
Pittsburgh Sleep Quality Index^g^	9.15 (2.53)	4-15	7.00-11.00	9.90 (2.80)	5-16	8.00-11.00
Polysomnography						
Sleep onset latency, min	16.13 (23.16)	1.0-162.5	5.50-17.95	13.56 (9.91)	1.0-53.0	6.25-18.00
Wake after sleep-onset, min	40.24 (31.99)	1.5-144.9	16.40-49.30	35.57 (30.77)	1.0-172.0	15.5-45.75
Early morning awakening, min	5.77 (10.03)	0-59.0	0.95-6.55	5.01 (6.55)	0-35.0	1.30-5.40
Time in bed, min	490.81 (74.29)	332-739	441.55-535.75	474.62 (68.49)	342.0-755.5	431.25-508.6
Total sleep time, min	434.43 (64.41)	300.5-605.0	383.50-475.50	425.48 (55.48)	297.0-560.5	386.75-474.00
Sleep efficiency, %	88.88 (7.38)	68.8-97.7	85.65-94.55	89.94 (5.77)	74.2-97.8	87.05-94.40
Non-REM sleep, %	86.29 (8.37)	70.70-119.20	81.60-91.50	83.94 (6.43)	67.20-100.00	79.45-88.60
REM sleep, %	14.23 (7.20)	0.3-29.4	9.25-18.40	16.05 (6.43)	0-32.8	11.35-20.55
Arousal, No./h	4.48 (2.15)	0.5-12.2	1.10-7.35	4.62 (2.35)	0.7-11.5	2.85-5.65
**Other variables**						
Hospital Anxiety and Depression Scale^h^						
Anxiety	8.98 (3.72)	1-17	6.00-12.00	8.54 (3.52)	2-18	6.00-10.00
Depressive symptoms	5.07 (9.77)	0-14	2.00-7.00	5.26 (3.50)	0-15	2.50-8.00
SF-36^i^						
Mental component	254.88 (73.98)	63-381	43.36-54.05	255.04 (76.96)	64-359	198.83-315.25
Physical component	242.82 (63.07)	102.5-385	203.13-283.75	220.70 (70.36)	77.5-355	166.25-282.50
Physical activity, %						
Sedentary	48.47 (7.68)	30.87-70.70	44.35-51.51	49.21 (6.55)	37.11-68.55	44.35-51.51
Light	39.63 (6.34)	22.93-55.06	35.04-43.18	38.99 (5.43)	20.95-48.65	35.90-42.27
Moderate	11.91 (4.14)	3.09-23.77	8.78-14.46	11.79 (4.18)	4.78-23.07	8.91-14.57
Vigorous	0	0	0	0	0	0
Very vigorous	0	0	0	0	0	0
MVPA	11.91 (4.14)	3.09-23.77	8.78-14.46	11.79 (4.18)	4.78-23.07	8.91-14.57
Step count	13581.19 (3393.02)	6049.86-21467.14	11 321.29–15 572.39	13 153.52 (2822.62)	8039.00-18 777.71	11 138.14–15 303.36

Abbreviations: BEPM, best evidence pain management; BMI, body mass index (calculated as weight in kilograms divided by height in meters squared); CBTi, cognitive behavioral therapy for insomnia; DBAS, Dysfunctional Beliefs and Attitudes About Sleep; kgf, kilogram force; MVPA, moderate-to-vigorous physical activity; NA, not applicable; REM, rapid eye movement; SF-36, 36-item Short-Form Health Survey.

^a^ Possible score range, 0 to 10. Higher scores indicate higher pain intensity or interference.

^b^ Possible score range, 0 to 100. Higher scores indicate higher changes of symptoms related to central sensitization; cutoff is set at 40 of 100.

^c^ Possible score range, 0 to 12. Higher scores indicate higher subjective levels of fatigue.

^d^ Possible score range, 0 to 10. A higher score indicates more dysfunctional beliefs and attitudes about sleep.

^e^ Possible score range, 0 to 24. Results ranging from 0 to 10 show average (normal) daytime sleepiness, while scores of 11 to 24 indicate excessive (abnormal) daytime sleepiness.

^f^ Possible score range, 0 to 28. Score of 0 to 7 indicates no clinically significant insomnia, 8 to 14 indicates subthreshold insomnia, 15 to 21 indicates moderate clinical insomnia, and 22 to 28 indicates severe clinical insomnia.

^g^ Possible score range, 0 to 21. A higher score indicates poorer sleep quality.

^h^ Possible score range, 0 to 21. Higher scores indicate a higher chance of anxiety or depressive symptoms.

^i^ Possible score range, 0 to 400. Higher scores indicate a better health condition.

**Table 2.** Dominant Pain Problem, Sex, and Education Variables

Variable	Frequencies, n (%)	
	BEPT only (n = 61)	BEPT with CBTi (n = 62)
Dominant pain problem		
Neck pain	36 (56.5)	35 (57.4)
Low back pain	27 (43.5)	26 (42.6)
Sex		
Male	21 (33.9)	20 (32.8)
Female	41 (66.1)	41 (67.2)
Educational level		
Master’s degree	28 (45.2)	22 (36.1)
Bachelor’s degree	24 (38.7)	23 (37.7)
Higher secondary education	10 (16.1)	15 (24.6)
Lower secondary education	0	1 (1.6)

Abbreviations: BEPT, best-evidence physical therapy; CBTi, cognitive behavioral therapy for insomnia.

### Effect of the Interventions

Table 3 and Table 4 present the results of the analyses using change values. A visual representation of these results is additionally provided in eFigures 1-4 in Supplement 2. For analyses with absolute scores, see eTable 8 in Supplement 2.

**Table 3.** Clinical Effectiveness Outcomes: Pain

Outcome^a^	Change time point^b^	Estimated margin, mean (SE)		Mean group difference (95% CI)	*P* value	Effect size, Cohen *d*
		CBTi-BEPM (n = 61)	BEPM (n = 62)			
**Primary outcome, mean pain intensity**						
Brief Pain Inventory, mean pain intensity	1	1.667 (0.331)	1.352 (0.331)	0.315 (−0.614 to 1.244)	.50	0.121
	2	1.407 (0.361)	1.093 (.0361)	0.315 (−0.697 to 1.327)	.54	0.111
	3	1.743 (0.309)	1.370 (0.304)	0.373 (−0.487 to 1.233)	.39	0.155
	4	1.976 (0.368)	1.006 (0.360)	0.970 (−0.051 to 1.992)	.06	0.340
**Secondary pain-related outcomes**						
Brief Pain Inventory						
Worst pain intensity	1	1.222 (0.268)	0.926 (0.268)	0.296 (−0.455 to 1.048)	.44	0.141
	2	0.926 (0.301)	1.130 (0.301)	−0.204 (−1.049 to 0.641)	.63	0.086
	3	1.399 (0.308)	1.130 (0.303)	0.270 (−0.588 to 1.127)	.54	0.112
	4	1.105 (0.308)	0.994 (0.297)	0.112 (−0.737 to 0.961)	.79	0.047
Least pain intensity	1	0.907 (0.240)	0.741 (0.240)	0.167 (−0.507 to 0.841)	.63	0.088
	2	1.000 (0.232)	1.074 (0.232)	−0.074 (−0.724 to 0.576)	.82	0.041
	3	0.996 (0.263)	0.778 (0.260)	0.219 (−0.514 to 0.952)	.56	0.106
	4	1.404 (0.255)	0.938 (0.250)	0.466 (−0.241 to 1.173)	.20	0.235
Pain intensity now	1	1.704 (0.347)	0.852 (0.347)	0.852 (−0.120 to 1.824)	.09	0.313
	2	1.259 (0.348)	1.241 (0.348)	0.019 (−0.958 to 0.995)	.97	0.007
	3	1.618 (0.322)	0.944 (0.317)	0.674 (−0.223 to 1.570)	.14	0.269
	4	1.642 (0.371)	0.949 (0.359)	0.692 (−0.332 to 1.716)	.18	0.242
Pain severity	1	1.375 (0.232)	0.968 (0.232)	0.407 (−0.243 to 1.058)	.22	0.223
	2	1.148 (0.250)	1.134 (0.250)	0.014 (−0.687 to 0.715)	.97	0.007
	3	1.438 (0.232)	1.056 (0.229)	0.382 (−0.264 to 1.029)	.24	0.211
	4	1.535 (0.364)	0.966 (0.257)	0.570 (−0.162 to 1.301)	.13	0.231
Pain interference^c^	1	1.651 (0.282)	0.910 (0.282)	0.741 (−0.050 to 1.531)	.07	0.335
	2	1.788 (0.271)	0.944 (0.271)	0.844 (0.084 to 1.604)	.03	0.397
	3	1.761 (0.273)	1.011 (0.269)	0.750 (−0.010 to 1.511)	.05	0.353
	4	1.771 (0.277)	1.063 (0.270)	0.708 (−0.060 to 1.475)	.07	0.330
Central sensitization inventory	1	12.981 (1.239)	7.481 (1.239)	5.500 (2.026 to 8.974)	.002	0.566
	2	10.648 (1.332)	8.796 (1.332)	1.852 (−1.884 to 5.588)	.33	0.177
	3	10.931 (1.480)	6.185 (1.464)	4.746 (0.615 to 8.877)	.03	0.411
	4	11.033 (1.487)	7.482 (1.454)	3.551 (−0.574 to 7.676)	.09	0.308
Pressure pain thresholds, kgf						
Primary	1	−1.249 (00.232)	−0.691 (0.235)	−0.557 (−1.211 to 0.097)	.09	0.305
	4	−1.171 (0.290)	−1.229 (0.273)	0.058 (−0.733 to 0.849)	.89	0.026
Calf, secondary	1	−0.526 (0.183)	−0.336 (0.185)	−0.161 (−0.677 to 0.355)	.54	0.132
	4	−0.844 (0.251)	−0.813 (0.240)	−0.031 (−0.719 to 0.657)	.93	0.016
Hand, secondary	1	−0.591 (0.143)	−0.187 (0.145)	−0.403 (−0.806 to −0.001)	.05	0.358
	4	−0.869 (0.188)	−0.704 (0.176)	−0.165 (−0.677 to 0.347)	.52	0.116

Abbreviations: BEPM, best-evidence pain management; CBTi-BEPM, cognitive behavioral therapy for insomnia integrated in BEPM; kgf, kilogram force.

^a^ Scoring scales for the tests are reported in the Table 1 footnotes.

^b^ Change 1 = baseline – time point 1 (immediate post-intervention); change 2 = baseline – time point 2 (3 months post-intervention); change 3 = baseline – time point 3 (6 months postintervention); change 4 = baseline – time point 4 (12 months postintervention, primary end point).

^c^ Due to baseline differences in Brief Pain Inventory (BPI) pain interference between groups, a subsample was defined to reach similar baseline scores. This was done to determine whether the reported group difference in BPI interference was due to the intervention received or the fact that the CBTi-BEPM group started with a higher mean BPI pain interference level. The 10% highest scores on the baseline BPI values were excluded from the dataset (12 exclusions from the CBTi-BEPM group and 3 exclusions from the BEPM-only group), resulting in equal groups at baseline for BPI pain interference. There was still a significant main effect for group (*P* = .30) with post hoc analysis, but only a significant difference for change 3 (T0 – T3; *P* = .04).

**Table 4.** Clinical Effectiveness Outcomes: Sleep, Psychiatric, and Physical Activity

Outcome^a^	Change time point^b^	Estimated margin, mean (SE)		Mean group difference (95% CI)	*P* value	Effect size, Cohen *d*
		CBTi-BEPM (n = 61)	BEPM (n = 62)			
**Secondary sleep-related outcomes**							
Insomnia Severity Index	1	8.296 (0.622)	2.722 (0.622)	5.574 (3.829 to 7.319)	<.001	1.143
	2	8.019 (0.687)	3.852 (0.687)	4.167 (2.241 to 6.092)	<.001	0.773
	3	8.075 (0.724)	3.537 (0.714)	4.538 (2.520 to 6.557)	<.001	0.805
	4	7.350 (0.750)	4.154 (0.730)	3.197 (1.121 to 5.273)	.003	0.551
Epworth Sleepiness Scale	1	1.167 (0.412)	0.352 (0.412)	0.815 (−0.339 to 1.969)	.17	0.252
	2	1.407 (0.511)	1.074 (0.511)	0.333 (−1.098 to 1.765)	.65	0.083
	3	1.662 (0.523)	1.167 (0.515)	0.495 (−0.961 to 1.951)	.50	0.112
	4	1.400 (0.504)	1.269 (0.492)	0.131 (−1.266 to 1.529)	.85	0.034
Pittsburgh Sleep Quality Index	1	4.667 (0.455)	1.907 (0.455)	2.759 (1.484 to 4.035)	<.001	0.774
	2	4.537 (0.402)	2.685 (0.402)	1.852 (0.723 to 2.980)	.002	0.460
	3	4.265 (0.461)	2.352 (0.453)	1.913 (0.631 to 3.195)	.004	0.534
	4	4.029 (0.462)	2.363 (0.450)	1.666 (0.387 to 2.944)	.01	0.466
Brugmann Fatigue Scale						
Mental	1	0.926 (0.302)	0.944 (0.302)	−0.019 (−0.864 to 0.827)	.97	0.008
	2	1.148 (0.325)	1.222 (0.325)	−0.074 (−0.895 to 0.837)	.87	0.029
	3	1.220 (0.353)	0.926 (0.349)	0.294 (−0.692 to 1.279)	.56	0.107
	4	0.943 (0.347)	1.038 (0.341)	−0.095 (−1.060 to 0.871)	.85	0.035
Physical	1	1.000 (0.243)	0.315 (0.243)	0.685 (0.004 to 1.366)	.05	0.359
	2	1.204 (0.260)	0.426 (0.260)	0.778 (0.048 to 1.508)	.04	0.382
	3	1.301 (0.293)	0.204 (0.289)	1.098 (0.282 to 1.914)	.009	0.481
	4	1.331 (0.250)	0.635 (0.243)	0.696 (0.005 to 1.387)	.05	0.360
DBAS	1	0.703 (0.067)	0.257 (0.067)	0.446 (0.259 to 0.633)	<.001	0.849
	2	0.745 (0.071)	0.312 (0.071)	0.433 (0.233 to 0.633)	<.001	0.778
	3	0.792 (0.071)	0.362 (0.070)	0.430 (0.231 to 0.628)	<.001	0.778
	4	0.754 (0.069)	0.392 (0.067)	0.362 (0.172 to 0.553)	<.001	0.679
PSG						
Sleep-onset latency, min	1	1.467 (1.830)	2.873 (1.891)	−1.406 (−6.625 to 3.813)	.59	.096
	4	1.747 (3.599)	−4.262 (3.405)	6.009 (−3.839 to 15.858)	.23	0.219
Wake after sleep onset, min	1	2.972 (5.285)	14.457 (5.426)	−11.485 (−26.509 to 3.539)	.13	0.273
	4	−4.967 (7.784)	7.036 (7.267)	−12.003 (−33.202 to 9.196)	.26	.203
Early morning awakenings, min	1	−0.726 (1.937)	−2.857 (1.989)	2.130 (−3.376 to 7.637)	.45	.138
	4	−2.699 (3.625)	−7.705 (3.378)	5.006 (−4.860 to 14.873)	.32	.182
Time in bed, min	1	8.080 (11.035)	16.010 (11.358)	−7.930 (−39.332 to 23.472)	.62	.090
	4	−16.633 (12.985)	0.994 (12.502)	−17.627 (−53.413 to 18.159)	.33	.176
Total sleep time, min	1	9.106 (10.636)	7.832 (10.861)	1.274 (−28.874 to 31.422)	.93	.015
	4	−4.456 (12.967)	12.986 (12.379)	−17.441 (−53.068 to 18.203)	.33	.176
Sleep efficiency, %	1	0.507 (1.227)	−1.154 (1.258)	1.660 (−1.825 to 5.146)	.35	.170
	4	2.256 (1.685)	2.394 (1.582)	−0.138 (−4.738 to 4.462)	.95	.011
REM sleep, %	1	−4.266 (1.084)	−5.069 (1.110)	0.803 (−2.274 to 3.880)	.61	.093
	4	−3.480 (1.229)	−5.398 (1.161)	1.918 (−1.448 to 5.283)	.26	.205
Non-REM sleep, %	1	4.258 (1.085)	5.056 (1.111)	−0.798 (−3.878 to 2.282)	.61	.093
	4	3.477 (1.229)	5.384 (1.161)	−1.907 (−5.274 to 1.460)	.26	.204
Arousal, No. of events	1	−3.891 (0.729)	−3.971 (0.750)	0.080 (−1.993 to 2.154)	.94	.014
	4	−5.854 (1.143)	−5.226 (1.058)	−0.628 (−3.732 to 2.476)	.69	.073
**Other secondary outcomes**							
SF-36						
Mental	1	−43.725 (10.162)	−33.151 (10.162)	−10.574 (−39.065 to 17.917)	.46	.133
	2	−51.019 (10.642)	−36.769 (10.642)	−14.250 (−44.087 to 15.587)	.35	.171
	3	−42.491 (9.906)	−27.182 (9.732)	−15.309 (−42.848 to 12.230)	.27	.199
	4	−37.722 (10.522)	−33.979 (10.239)	−3.743 (−32.880 to 25.395)	.80	.046
Physical	1	−73.796 (9.964)	−56.620 (9.964)	−17.176 (−45.113 to 10.762)	.23	.220
	2	−69.398 (10.735)	−51.481 (10.735)	−17.917 (−48.016 to 12.183)	.24	.213
	3	−60.811 (10.072)	−43.889 (9.833)	−16.922 (−44.771 to 10.927)	.23	.217
	4	−73.935 (9.421)	−48.990 (9.193)	−24.945 (−51.044 to 1.155)	.06	.342
Hospital Anxiety and Depression Scale						
Anxiety	1	2.519 (0.419)	1.741 (0.419)	0.778 (−0.396 to 1.952)	.19	.237
	2	2.315 (0.463)	2.000 (0.463)	0.315 (−0.984 to 1.613)	.63	.089
	3	2.549 (0.492)	1.222 (0.485)	1.326 (−0.044 to 2.697)	.06	.346
	4	2.604 (0.444)	1.686 (0.432)	0.918 (−0.311 to 2.148)	.14	.267
Depression	1	2.259 (0.372)	1.167 (0.372)	1.093 (0.050 to 2.135)	.04	.374
	2	2.148 (0.400)	1.037 (0.400)	1.111 (−0.010 to 2.232)	.05	.354
	3	2.006 (0.384)	0.722 (0.380)	1.283 (.213 to 2.354)	.02	.429
	4	2.291 (0.215)	1.336 (0.406)	0.955 (−0.196 to 2.106)	.10	.373
Physical activity, %						
Sedentary	1	−1.092 (0.894)	−0.006 (0.908)	1.086 (−1.443 to 3.615)	.40	.154
	4	−0.746 (1.017)	−0.192 (1.015)	0.553 (−2.307 to 3.414)	.70	.070
Light	1	0.686 (0.823)	0.693 (0.838)	0.007 (−2.324 to 2.338)	.99	.001
	4	0.222 (0.880)	−0.004 (0.880)	−0.226 (−2.705 to 2.254)	0.86	.034
Moderate	1	0.421 (0.423)	−0.664 (0.427)	−1.085 (−2.277 to 0.108)	0.07	.326
	4	0.507 (0.392)	0.223 (0.390)	−0.284 (−1.384 to 0.816)	0.61	.093
Moderate/vigorous	1	0.421 (0.423)	−0.664 (0.427)	−1.085 (−2.277 to 0.108)	.07	.326
	4	0.507 (0.392)	0.223 (0.390)	−0.284 (−1.384 to 0.816)	.61	.093
Step count	1	456.670 (349.893)	−230.904 (352.699)	−687.574 (−1673.244 to 298.096)	.20	.250
	4	124.221 (337.101)	504.398 (335.763)	380.177 (−565.523 to 1325.877)	.43	.144

Abbreviations: BEPM, best-evidence pain management; CBTi-BEPM, cognitive behavioral therapy for insomnia integrated in BEPM; DBAS, Dysfunctional Beliefs and Attitudes About Sleep; PSG, polysomnography; REM, rapid eye movement; SF-36, 36-item Short-Form Health Survey.

^a^ Scoring scales for the tests are reported in the Table 1 footnotes.

^b^ Change 1 = baseline – time point 1 (immediate post-intervention); change 2 = baseline – time point 2 (3 months post-intervention); change 3 = baseline – time point 3 (6 months postintervention); change 4 = baseline – time point 4 (12 months postintervention, primary end point).

Analysis of the primary clinical outcome, ie, differences in mean pain intensity (BPI) at 12-month following intervention, indicates no significant difference was observed between the 2 groups, with an MGD of 0.970 points (95% CI, −0.051 to 1.992; effect size Cohen *d*, 0.340) (Table 3). In 99 participants (80.5%) with 12-month BPI data, the mean pain intensity at 12 months decreased by 1.976 points (reduction of 40%) in the CBTi-BEPM group and 1.006 points (reduction of 24%) points in the BEPM-only group. Similarly, the other time points showed small, nonsignificant differences (MGD ranging from 0.315 points; 95% CI, −0.614 to 1.244; to 0.373; 95% CI, −0.487 to 1.233 points). From a clinical perspective, CBTi-BEPM resulted in a treatment response for BPI-measured mean pain intensity with a number needed to treat (NNT) of 4 (95% CI, 2-6) observed over a period of 12 months (NNT directly posttreatment was 14; 95% CI, 7-19). On a preliminary secondary basis, similar analyses were performed for other pain-related outcomes, sleep-related outcomes, physical activity, depressive symptoms and anxiety, and QOL (Table 3 and Table 4).

For pain interference (BPI), CBTi-BEPM showed a significantly greater change from baseline to 3-month follow-up compared with BEPM only (MGD: 0.844 points: 95% CI 0.084-1.604; small effect [Cohen *d*]). While no significant results were found for BPI pain severity and pain intensity now, only the percent within-group-change of the CBTi-BEMP group exceeded the MCID at 12-month follow-up (severity: −33%, pain intensity: 39% in CBTi-BEPM vs severity: −23%, pain intensity: 27% in BEPM only). For the central sensitization inventory, CBTi-BEPM showed a significantly greater improvement from baseline to directly after treatment (MGD: 5.500 points 95% CI, 2.026-8.974 points; medium effect) as well as to 6-month follow-up (MGD: 4.746 points; 95% CI, 0.615-8.877 points; small effect) compared with BEPM only. For the Insomnia Severity Index, compared with BEPM only, CBTi-BEPM resulted in a larger reduction of insomnia severity from baseline to directly after intervention (MGD: 5.574 points; 95% CI, 3.829-7.319 points; large effect) and to 3 months (MGD: 4.167 points; 95% CI, 2.241-6.092 points; medium effect), 6 months (MGD: 4.538 points; 95% CI, 2.520-6.557 points; large effect), and 12 months (MGD: 3.197 points; 95% CI, 1.121-5.273; medium effect) after intervention. Effect sizes ranged from 0.551 to 1.143. Response and remission analyses for insomnia were based on the MCID (6 points) and cutoff value (14 of 28) on the Insomnia Severity Index. Directly after treatment, 79.6% of participants in the CBTi-BEPM group responded and 90.1% achieved remission, compared with 37.0% who responded and 70.4% who achieved remission in the BEPM-only group. At the 12-month follow-up, responder rates remained high at 63.8% and remission at 87.2% in the CBTi-BEPM group, compared with 38.5% response and 78.8% remission in the BEPM-only group. Based on these rates, NNTs were calculated. The NNT for achieving insomnia remission with CBTi-BEPM was 5 (95% CI, 4-6) directly after treatment and 12 (95% CI, 8-16) after 12-month follow-up. With regard to treatment response (based on Insomnia Severity Index MCID), the NNT was 2 (95% CI, 1-3) directly after treatment and 4 (95% CI, 3-5) at 12-month follow-up. Full details are presented in eTable 6 in Supplement 2.

Similarly, a significantly greater improvement was found after CBTi-BEPM vs BEPM only from baseline to all postintervention time points for the Dysfunctional Beliefs and Attitudes About Sleep questionnaire (medium to large effects), the Pittsburgh Sleep Quality Index (small to medium effects), and the Brugmann Fatigue Scale - Physical Fatigue (small effects)For the Depressive Symptom subscale of the Hospital Anxiety and Depression Rating Scale, CBTi-BEPM showed a significantly greater improvement from baseline to directly after treatment (MGD: 1.093 points; 95% CI, 0.050-2.135; small effect) as well as to 6-month follow-up (MGD: 1.283 points; 95% CI, 0.213-2.354; small effect) compared with BEPM only. Changes in depression exceeded the MCID of 1.7 points. However, initial levels at baseline decreased beneath the threshold of 7 of 21, which typically denotes the presence of depressive symptoms. All other outcomes showed no significant group differences.

No serious adverse events were reported. One participant developed a cervical disc herniation, which led to an increase in primary nociceptive pain, and the patient discontinued the trial.

### Sensitivity Analysis

Comprehensive results of the sensitivity analysis (ie, adjusted for baseline pain and insomnia levels) are provided in eTable 4 in Supplement 2 with 2 notable findings. First, the previously observed significant difference favoring CBTi-BEPM over the control group in pain interference (BPI) from baseline to 3-month follow-up was no longer evident (MGD, 0.555; 95% CI, 1.280-0.170; *P* = .13). Additionally, for the Brugmann Fatigue Scale physical dimension—across all time points, except for the change from baseline to 6-month follow-up (MGD, 0.929; 95% CI, 1.759-0.099; *P* = .03)—the previously noted significant difference in favor of the CBTi-BEPM group was no longer present (MGD range, 0.388-0.658; *P* < .09).

Regarding the second sensitivity analysis (ie, baseline difference between dropout and no dropout), except for age (with a slightly younger age in the dropout group) no significant baseline differences were found. eTable 5 in Supplement 2 presents detailed results.

## Discussion

Our hypothesis that CBTi-BEPM in patients with nCSP and insomnia would lead to larger improvements in mean pain intensity at 12-month follow-up, compared with BEPM only, was not confirmed. This finding aligns with trials evaluating the efficacy of CBTi in other pain populations.^19,51,52,53,54^ These results may stem from including the BEPM-only treatment as a control intervention, which offers high-quality, individualized care and substantial pain relief across a wider nCSP population.^50,55^ The relatively smaller improvements observed for CBTi-BEPM vs BEPM only are therefore not an indication of an ineffective therapy. Both intervention groups did not receive equal dosing of BEPM (9 vs 15 sessions). It is possible that the minimal group differences for mean pain intensity were the result of this different dosing in active pain treatment. It is noteworthy that pain outcomes did not differ significantly among patients with nCSP and insomnia in the integrated group receiving a reduced dose of BEPM, although some methodologic aspects warrant consideration when seeking to clarify these relatively minor differences in mean pain intensity. The selected primary outcome, mean pain intensity in the last 24 hours (BPI), might not be entirely fit for the sleep-pain association. A single 24-hour measurement of mean pain intensity may inadequately capture the effects of insomnia symptom changes and could be highly sensitive to proximal events. Assessing pain outcomes over a 1- to 2-week period concurrent with sleep measures could offer more valid evaluation of their association, potentially yielding different pain-related results. Additionally, including participants with higher baseline average pain levels might have led to different outcomes at 12-month follow-up. Our pain intensity findings suggest susceptibility to floor effects, given the relatively low baseline mean score and the requisite 30% change for clinical significance.

On a preliminary basis, CBTi-BEPM was, consistently over the different time points and different analyses, more effective than BEPM only for improving insomnia severity, sleep quality, and unhelpful beliefs about sleep, which is in line with similar research in other pain populations.^19,51,52,53,54^ At 12-month follow-up, the CBTi-BEPT group also decreased below the threshold for clinical insomnia, which was not seen in the BEPM-only group. This is noteworthy because insomnia has a strong negative effect on QOL and is further predictive of neurologic, immune, and cardiovascular disorders. Also preliminary, CBTi-BEPM was more effective in improving depressive symptoms, again in line with other research in different pain populations.^53,54^

We found no effects on PSG sleep outcomes, indicating that CBTi has an added value to impact subjective but not objective sleep. Yet, this study only assessed PSG outcomes of sleep quantity and sleep macroarchitecture, and methodologic considerations (ie, only 1 night of assessment) might also clarify the nonsignificant results in PSG. In the context of the highly variable nature of sleep, future research might consider measuring sleep over an extended period, which will likely offer a more ecologically valid assessment by capturing the natural sleep fluctuations over time. Reliable contemporary electroencephalogram sleep monitoring devices that are less invasive than PSG and easily applicable for at-home recording over multiple nights are accessible.^56,57^ As such, future in-depth assessments at microarchitecture level (eg, electroencephalogram spectral power analysis, analysis of cyclic alternating patterns) might lead to different outcomes when comparing CBTi-BEPM with BEMP-only.

In addition, objective PSG outcomes show that only the borderline diagnostic cutoff for insomnia was reached for waking after sleep onset, while the duration of objective sleep-onset latency and early-morning awakenings was minimally affected in both groups (<17 minutes). Including participants with significant levels of clinical insomnia as measured by PSG might lead to different results for the sleep outcomes. However, given the favorable outcomes of CBTi-BEPM on self-reported sleep, this intervention is certainly advised in persons with mild or subclinical insomnia.

### Clinical Interpretation of Response

Given the detrimental effects of insomnia,^11^ clinical implementation of CBTi-BEPM is advised even in the absence of an overall large additional effect on pain intensity. While no statistically significant differences were observed, interpreting the results within a clinical context considering meaningful within-group changes leads to several observations. First, for BPI mean pain intensity at 12-month follow-up (primary end point), the percent within-group change was clinically relevant for CBTi-BEPM (−40%), but not for BEPM only (−23%).^25^ Moreover, CBTi-BEPM resulted in a treatment response for BPI average pain with an NNT of 4 observed over a period of 12 months. Furthermore, for BPI pain severity and pain intensity now, only the percent within-group change of the CBTi-BEMP group exceeded the MCID (severity: −33%, pain intensity: 39% in CBTi-BEPM vs severity: −24%, pain intensity: 27% in BEPM only at 12-month follow-up). Regarding BPI least pain intensity and pressure pain threshold, both groups exceeded the MCID, which underscores the effectiveness of BEPM, and suggests that it extends to the highly disabled nCSP subgroup with insomnia.

Within-group changes in insomnia severity and sleep quality held clinical significance solely within the CBTi-BEPM group. The CBTi-BEPM intervention showed higher rates of people with response and remission for insomnia severity both immediately after treatment and at the 12-month mark. Directly after treatment, CBTi-BEPM showed a 79.6% response rate and 90.1% remission rate, compared with a 37% response rate and 70.4% remission rate in the BEPM-only group. Moreover, the NNT for achieving insomnia remission with CBTi-BEPM was 5 directly after treatment and 12 at 12-month follow-up, while for treatment responders, the NNT was 2 directly after treatment and 4 at 12-month follow-up. Concerning depressive symptoms, both groups consistently scored below the 7-point threshold on the Hospital Anxiety and Depression Rating Scale–Depressive symptoms subscale across all postintervention time points. However, it was observed that only the CBTi-BEPM group exceeded the MCID at all follow-up assessments. Specifically for anxiety, assessed using the same questionnaire, both intervention groups exceeded the MCID after intervention, which was sustained at long-term follow-up (ie, 1.7-point decrease). Nevertheless, only the CBTi-BEPM group decreased below the cutoff level (ie, 7 of 21).^41,42^

### Strengths and Limitations

This study has several strengths. To our knowledge, this is the first triple-blind, multicenter, fully powered randomized clinical trial examining the treatment effects of CBTi-BEPM in patients with nCSP and insomnia, with an a priori published trial^50^ and intervention^46^ protocol and long-term follow-up. Participants were objectively screened via PSG on underlying intrinsic sleep disorders. The trial used BEPM as a high-quality control intervention within balanced treatment arms and therapists provided either the experimental or control treatment to minimize nonspecific treatment effects and optimize external validity. The number of sessions complied with standard Belgian physical therapy reimbursement, facilitating implementation. Treatment fidelity was verified during follow-up refresher sessions to avoid therapy drift. Moreover, therapeutic alliance was checked twice during the intervention period (after weeks 2 and 8), using an online questionnaire. Since both interventions include person-centered approaches, self-management strategies, individual goal setting, and adaptations based on level of behavioral change as key elements, adherence to the personalized treatment plan was closely monitored by the therapists. Additionally, sleep diaries tracked adherence the CBTi in the experimental group.

Some limitations should be considered. Persons with obesity or depression were excluded, resulting in a specific subsample of the population, limiting generalizability. The data analysis encompassed a substantial number of outcomes (ranging from 2 to 4 time points depending on the outcome and 34 outcomes). Yet, it did not implement correction for multiple comparison, a decision grounded in consideration of the sample size and the prioritization of minimizing type II errors. Consequently, significant results should be interpreted with caution, which led to our emphasis on patterns and magnitudes of effects, transcending mere statistical significance. Given the nature of the intervention, the treating physiotherapists were not blinded. However, assessors and statisticians were blinded. In addition, COVID-19 and technical issues resulted in missing data. However, the 20% expected dropout rate embedded in the sample size analysis was not exceeded.

## Conclusions

In this randomized clinical trial, CBTi-BEPM showed no statistically significant effect on mean pain intensity (primary outcome). Yet, on a preliminary basis, CBTi-BEPM led to improving insomnia severity, sleep quality, beliefs about sleep, depressive symptoms, and physical fatigue. Changes in insomnia severity and sleep quality were clinically meaningful. Given the detrimental effects of insomnia on daytime functioning and QOL, CBTi should be integrated in BEPM for patients with nCSP and insomnia.

## Notes

Supplement 1.
Trial Protocol and Statistical Analysis Plan

Supplement 2. **eTable 1.** Inclusion and Exclusion Criteria **eMethods.** Details on Polysomnography Assessment **eTable 2.** Training and Supervision of Therapists **eTable 3.** Overview of Missing Data per Outcome and per Time Point **eFigure 1.** Forest Plot Showing the Results of the Analyses Using Delta Values for Self-Reported Pain-Related Outcomes **eFigure 2.** Forest Plot Showing the Results of the Analyses Using Delta Values for Self-Reported Sleep-Related Outcomes **eTable 4.** Sensitivity Analyses **eFigure 3.** Forest Plot Showing the Results of the First Sensitivity Analyses Using Delta Values for Self-Reported Pain-Related Outcomes **eFigure 4.** Forest Plot Showing the Results of the First Sensitivity Analyses Using Delta Values for Self-Reported Sleep-Related Outcomes **eTable 5.** Second Sensitivity Analyses, Comparing Dropouts to Non-dropouts **eTable 6.** Remitters, Responders and NNT Analyses **eTable 7.** Success of Assessor and Participant Blinding **eTable 8.** Absolute Values at Each Time Point
eReferences

Supplement 3.
Data Sharing Statement

## References

[1] Hartvigsen J, Hancock MJ, Kongsted A, ; Lancet Low Back Pain Series Working Group. What low back pain is and why we need to pay attention. Lancet. 2018;391(10137):2356-2367. doi:10.1016/S0140-6736(18)30480-X 29573870

[2] Van Looveren E, Bilterys T, Munneke W, . The association between sleep and chronic spinal pain: a systematic review from the last decade. J Clin Med. 2021;10(17):3836. doi:10.3390/jcm10173836 34501283 PMC8432009

[3] Sateia MJ. International classification of sleep disorders-third edition: highlights and modifications. Chest. 2014;146(5):1387-1394. doi:10.1378/chest.14-0970 25367475

[4] Tang NKY, Wright KJ, Salkovskis PM. Prevalence and correlates of clinical insomnia co-occurring with chronic back pain. J Sleep Res. 2007;16(1):85-95. doi:10.1111/j.1365-2869.2007.00571.x 17309767

[5] Artner J, Cakir B, Spiekermann JA, . Prevalence of sleep deprivation in patients with chronic neck and back pain: a retrospective evaluation of 1016 patients. J Pain Res. 2013;6:1-6. 23300350 10.2147/JPR.S36386 PMC3536352

[6] Marin R, Cyhan T, Miklos W. Sleep disturbance in patients with chronic low back pain. Am J Phys Med Rehabil. 2006;85(5):430-435. doi:10.1097/01.phm.0000214259.06380.79 16628150

[7] McCracken LM, Iverson GL. Disrupted sleep patterns and daily functioning in patients with chronic pain. Pain Res Manag. 2002;7(2):75-79. doi:10.1155/2002/579425 12185371

[8] Daly-Eichenhardt A, Scott W, Howard-Jones M, Nicolaou T, McCracken LM. Changes in sleep problems and psychological flexibility following interdisciplinary acceptance and commitment therapy for chronic pain: an observational cohort study. Front Psychol. 2016;7:1326. doi:10.3389/fpsyg.2016.01326 27630601 PMC5006108

[9] Alsaadi SM, McAuley JH, Hush JM, Maher CG. Prevalence of sleep disturbance in patients with low back pain. Eur Spine J. 2011;20(5):737-743. doi:10.1007/s00586-010-1661-x 21190045 PMC3082679

[10] Bahouq H, Allali F, Rkain H, Hmamouchi I, Hajjaj-Hassouni N. Prevalence and severity of insomnia in chronic low back pain patients. Rheumatol Int. 2013;33(5):1277-1281. doi:10.1007/s00296-012-2550-x 23124732

[11] Sezgin M, Hasanefendioğlu EZ, Sungur MA, . Sleep quality in patients with chronic low back pain: a cross-sectional study assessing its relations with pain, functional status and quality of life. J Back Musculoskelet Rehabil. 2015;28(3):433-441. doi:10.3233/BMR-140537 25322735

[12] Finan PH, Goodin BR, Smith MT. The association of sleep and pain: an update and a path forward. J Pain. 2013;14(12):1539-1552. doi:10.1016/j.jpain.2013.08.007 24290442 PMC4046588

[13] Smith MT, Quartana PJ, Okonkwo RM, Nasir A. Mechanisms by which sleep disturbance contributes to osteoarthritis pain: a conceptual model. Curr Pain Headache Rep. 2009;13(6):447-454. doi:10.1007/s11916-009-0073-2 19889286

[14] Henschke N, Ostelo RW, van Tulder MW, . Behavioural treatment for chronic low-back pain. Cochrane Database Syst Rev. 2010;2010(7):CD002014. 20614428 10.1002/14651858.CD002014.pub3 PMC7065591

[15] Williams ACC, Fisher E, Hearn L, Eccleston C. Psychological therapies for the management of chronic pain (excluding headache) in adults. Cochrane Database Syst Rev. 2020;8(8):CD007407. 32794606 10.1002/14651858.CD007407.pub4 PMC7437545

[16] Perlis ML, Posner D, Riemann D, Bastien CH, Teel J, Thase M. Insomnia. Lancet. 2022;400(10357):1047-1060. doi:10.1016/S0140-6736(22)00879-0 36115372

[17] Riemann D, Baglioni C, Bassetti C, . European guideline for the diagnosis and treatment of insomnia. J Sleep Res. 2017;26(6):675-700. doi:10.1111/jsr.12594 28875581

[18] Edinger JD, Arnedt JT, Bertisch SM, . Behavioral and psychological treatments for chronic insomnia disorder in adults: an American Academy of Sleep Medicine clinical practice guideline. J Clin Sleep Med. 2021;17(2):255-262. doi:10.5664/jcsm.8986 33164742 PMC7853203

[19] Tang NK, Lereya ST, Boulton H, Miller MA, Wolke D, Cappuccio FP. Nonpharmacological treatments of insomnia for long-term painful conditions: a systematic review and meta-analysis of patient-reported outcomes in randomized controlled trials. Sleep. 2015;38(11):1751-1764. doi:10.5665/sleep.5158 25902806 PMC4813361

[20] McCurry SM, Zhu W, Von Korff M, . Effect of telephone cognitive behavioral therapy for insomnia in older adults with osteoarthritis pain: a randomized clinical trial. JAMA Intern Med. 2021;181(4):530-538. doi:10.1001/jamainternmed.2020.9049 33616613 PMC7900930

[21] Tang NK, Sanborn AN. Better quality sleep promotes daytime physical activity in patients with chronic pain? a multilevel analysis of the within-person relationship. PLoS One. 2014;9(3):e92158. doi:10.1371/journal.pone.0092158 24667276 PMC3965418

[22] Jungquist CR, O’Brien C, Matteson-Rusby S, . The efficacy of cognitive-behavioral therapy for insomnia in patients with chronic pain. Sleep Med. 2010;11(3):302-309. doi:10.1016/j.sleep.2009.05.018 20133188 PMC2830371

[23] Malfliet A, Bilterys T, Van Looveren E, . The added value of cognitive behavioral therapy for insomnia to current best evidence physical therapy for chronic spinal pain: protocol of a randomized controlled clinical trial. Braz J Phys Ther. 2019;23(1):62-70. doi:10.1016/j.bjpt.2018.10.007 30389347 PMC6546904

[24] Stanhope J. Brief Pain Inventory review. Occup Med (Lond). 2016;66(6):496-497. doi:10.1093/occmed/kqw041 27067913

[25] Dworkin RH, Turk DC, Wyrwich KW, . Interpreting the clinical importance of treatment outcomes in chronic pain clinical trials: IMMPACT recommendations. J Pain. 2008;9(2):105-121. doi:10.1016/j.jpain.2007.09.005 18055266

[26] Cuesta-Vargas AI, Neblett R, Chiarotto A, . Dimensionality and reliability of the central sensitization inventory in a pooled multicountry sample. J Pain. 2018;19(3):317-329. doi:10.1016/j.jpain.2017.11.006 29198933

[27] Kregel J, Vuijk PJ, Descheemaeker F, . The Dutch Central Sensitization Inventory (CSI): factor analysis, discriminative power, and test-retest reliability. Clin J Pain. 2016;32(7):624-630. doi:10.1097/AJP.0000000000000306 26418360

[28] Fischer A. Application of pressure algometry in manual medicine. J Man Med. 1990;5(4):145-150.

[29] Chesterton LS, Sim J, Wright CC, Foster NE. Interrater reliability of algometry in measuring pressure pain thresholds in healthy humans, using multiple raters. Clin J Pain. 2007;23(9):760-766. doi:10.1097/AJP.0b013e318154b6ae 18075402

[30] Cathcart S, Pritchard D. Reliability of pain threshold measurement in young adults. J Headache Pain. 2006;7(1):21-26. doi:10.1007/s10194-006-0265-7 16440140 PMC3451574

[31] Buysse DJ, Reynolds CF III, Monk TH, Berman SR, Kupfer DJ. The Pittsburgh Sleep Quality Index: a new instrument for psychiatric practice and research. Psychiatry Res. 1989;28(2):193-213. doi:10.1016/0165-1781(89)90047-4 2748771

[32] Alsaadi SM, McAuley JH, Hush JM, . Detecting insomnia in patients with low back pain: accuracy of four self-report sleep measures. BMC Musculoskelet Disord. 2013;14(1):196. doi:10.1186/1471-2474-14-196 23805978 PMC3701511

[33] Eadie J, van de Water AT, Lonsdale C, . Physiotherapy for sleep disturbance in people with chronic low back pain: results of a feasibility randomized controlled trial. Arch Phys Med Rehabil. 2013;94(11):2083-2092. doi:10.1016/j.apmr.2013.04.017 23643716

[34] Bastien CH, Vallières A, Morin CM. Validation of the Insomnia Severity Index as an outcome measure for insomnia research. Sleep Med. 2001;2(4):297-307. doi:10.1016/S1389-9457(00)00065-4 11438246

[35] Yang M, Morin CM, Schaefer K, Wallenstein GV. Interpreting score differences in the Insomnia Severity Index: using health-related outcomes to define the minimally important difference. Curr Med Res Opin. 2009;25(10):2487-2494. doi:10.1185/03007990903167415 19689221

[36] Blais FC, Gendron L, Mimeault V, Morin CM. Evaluation of insomnia: validity of 3 questionnaires. Article in French. Encephale. 1997;23(6):447-453. 9488928

[37] Morin CM, Vallières A, Ivers H. Dysfunctional Beliefs and Attitudes About Sleep (DBAS): validation of a brief version (DBAS-16). Sleep. 2007;30(11):1547-1554. doi:10.1093/sleep/30.11.1547 18041487 PMC2082102

[38] Johns MW. A new method for measuring daytime sleepiness: the Epworth sleepiness scale. Sleep. 1991;14(6):540-545. doi:10.1093/sleep/14.6.540 1798888

[39] Mairesse O, Damen V, Newell J, Kornreich C, Verbanck P, Neu D. The Brugmann Fatigue Scale: an analogue to the Epworth Sleepiness Scale to measure behavioral rest propensity. Behav Sleep Med. 2019;17(4):437-458. doi:10.1080/15402002.2017.1395336 29065269

[40] Bjelland I, Dahl AA, Haug TT, Neckelmann D. The validity of the Hospital Anxiety and Depression Scale: an updated literature review. J Psychosom Res. 2002;52(2):69-77. doi:10.1016/S0022-3999(01)00296-3 11832252

[41] Lemay KR, Tulloch HE, Pipe AL, Reed JL. Establishing the minimal clinically important difference for the hospital anxiety and depression scale in patients with cardiovascular disease. J Cardiopulm Rehabil Prev. 2019;39(6):E6-E11. doi:10.1097/HCR.0000000000000379 30489438

[42] Stern AF. The hospital anxiety and depression scale. Occup Med (Lond). 2014;64(5):393-394. doi:10.1093/occmed/kqu024 25005549

[43] Kwan RYC, Liu JYW, Lee D, Tse CYA, Lee PH. A validation study of the use of smartphones and wrist-worn ActiGraphs to measure physical activity at different levels of intensity and step rates in older people. Gait Posture. 2020;82:306-312. doi:10.1016/j.gaitpost.2020.09.022 33007688

[44] Lee P, Tse CY. Calibration of wrist-worn ActiWatch 2 and ActiGraph wGT3X for assessment of physical activity in young adults. Gait Posture. 2019;68:141-149. doi:10.1016/j.gaitpost.2018.11.023 30476691

[45] McHorney CA, Ware JE Jr, Raczek AE; Psychometric and Clinical Tests of Validity in Measuring Physical and Mental Health Constructs. The MOS 36-Item Short-Form Health Survey (SF-36): II. psychometric and clinical tests of validity in measuring physical and mental health constructs. Med Care. 1993;31(3):247-263. doi:10.1097/00005650-199303000-00006 8450681

[46] Van Looveren E, Meeus M, Cagnie B, . Combining cognitive behavioral therapy for insomnia and chronic spinal pain within physical therapy: a practical guide for the implementation of an integrated approach. Phys Ther. 2022;102(8):pzac075. doi:10.1093/ptj/pzac075 35689809

[47] Perlis MLBJC, Smith MT, Posner DA. Cognitive Behavioral Treatment of Insomnia: A Session-by-Session Guide. Springer New York; 2005.

[48] Nijs J, Mairesse O, Neu D, . Sleep disturbances in chronic pain: neurobiology, assessment, and treatment in physical therapist practice. Phys Ther. 2018;98(5):325-335. doi:10.1093/ptj/pzy020 29425327

[49] Malfliet A, Kregel J, Meeus M, . Applying contemporary neuroscience in exercise interventions for chronic spinal pain: treatment protocol. Braz J Phys Ther. 2017;21(5):378-387. doi:10.1016/j.bjpt.2017.06.019 28736211 PMC5628368

[50] Malfliet A, Kregel J, Coppieters I, . Effect of pain neuroscience education combined with cognition-targeted motor control training on chronic spinal pain: a randomized clinical trial. JAMA Neurol. 2018;75(7):808-817. doi:10.1001/jamaneurol.2018.0492 29710099 PMC6145763

[51] Vitiello MV, McCurry SM, Shortreed SM, . Cognitive-behavioral treatment for comorbid insomnia and osteoarthritis pain in primary care: the lifestyles randomized controlled trial. J Am Geriatr Soc. 2013;61(6):947-956. doi:10.1111/jgs.12275 23711168 PMC3772673

[52] Lami MJ, Martínez MP, Miró E, . Efficacy of combined cognitive-behavioral therapy for insomnia and pain in patients with fibromyalgia: a randomized controlled trial. Cognit Ther Res. 2018;42(1):63-79. doi:10.1007/s10608-017-9875-4

[53] Pigeon WR, Moynihan J, Matteson-Rusby S, . Comparative effectiveness of CBT interventions for co-morbid chronic pain & insomnia: a pilot study. Behav Res Ther. 2012;50(11):685-689. doi:10.1016/j.brat.2012.07.005 22982083 PMC3466363

[54] Tang NK, Goodchild CE, Salkovskis PM. Hybrid cognitive-behaviour therapy for individuals with insomnia and chronic pain: a pilot randomised controlled trial. Behav Res Ther. 2012;50(12):814-821. doi:10.1016/j.brat.2012.08.006 23123531

[55] Ashar YK, Gordon A, Schubiner H, . Effect of pain reprocessing therapy vs placebo and usual care for patients with chronic back pain: a randomized clinical trial. JAMA Psychiatry. 2022;79(1):13-23. doi:10.1001/jamapsychiatry.2021.2669 34586357 PMC8482298

[56] Stone JD, Rentz LE, Forsey J, . Evaluations of commercial sleep technologies for objective monitoring during routine sleeping conditions. Nat Sci Sleep. 2020;12:821-842. doi:10.2147/NSS.S270705 33149712 PMC7603649

[57] Arnal PJ, Thorey V, Debellemaniere E, . The Dreem Headband compared to polysomnography for electroencephalographic signal acquisition and sleep staging. Sleep. 2020;43(11):zsaa097. doi:10.1093/sleep/zsaa097 32433768 PMC7751170

