# Effect Size Errors in Abstract and Main Article Results, Table 3, Table 4, and Supplement 2

**DOI:** 10.1001/jamanetworkopen.2026.28732

**Published:** 2026-07-21

In the Original Investigation titled “Cognitive Behavioral Therapy for Insomnia in Pain Management for Nonspecific Chronic Spinal Pain: A Randomized Clinical Trial,”^1^ published August 9, 2024, there were effect size errors in the Abstract and main article Results, Table 3, Table 4, and Supplement 2. The automation process of the effect size calculation caused the error. The effect sizes have been recalculated and updated. The authors have explained what happened in a Comment,^2^ and this article has been corrected.^1^
