# Supplementary material for: Cognitive Behavioral Therapy for Insomnia in Pain Management for Nonspecific Chronic Spinal Pain: A Randomized Clinical Trial
Source: JAMA Netw Open. 2024 Aug 9;7(8):e2425856. doi: 10.1001/jamanetworkopen.2024.25856 (PMC11316234; doi:10.1001/jamanetworkopen.2024.25856)
Supplement: Supplement 1. — Trial Protocol and Statistical Analysis Plan [file jamanetwopen-e2425856-s001.pdf]

Supplementary material: Study protocol.

Original title:
The added value of cognitive behavioral therapy for insomnia to current best evidence physical therapy for chronic spinal pain: protocol of a randomized controlled clinical trial.

Objectives:
The main objective of this trial is to evaluate the effectiveness of cognitive behavioral therapy for insomnia integrated in best-evidence pain management (CBTi-BEPM) versus BEPM-only for reducing pain intensity at 12 months post-intervention. Additionally, on an exploratory basis, we will examine if CBTi-BEPM versus BEPM-only can improve other pain-related and sleep-related outcomes, physical activity, depressive symptoms and anxiety, and quality of life, extending up to 12-months after treatment.

Design:
The study comprised a multicentre, triple-blind, randomised, controlled, two-arm (1:1) trial. Treatment outcomes will be assessed at baseline, at post-intervention, and at 3, 6 and 12 months follow-up. At the 3 and 6-month follow-up, online self-reported outcome data was collected.

Study population:
One-hundred-twenty patients with nonspecific chronic spinal pain (nCSP) and comorbid insomnia will be recruited. Eligibility criteria are outlined in the table 1.

**Inclusion**	**Exclusion**
Nonspecific spinal pain for at least 3 months' duration, at least 3 days/week	Severe underlying sleep pathology (identified through baseline data of polysomnography)
Aged between 18 and 65 years	Neuropathic pain
Seeking care because of neck pain or low back pain	Chronic widespread pain syndromes
Native Dutch Speaker	Shift workers
Having insomnia: in the absence of other intrinsic sleep-disorders and shift work, insomnia is defined as > 30 min of sleep latency and/or minutes awake after sleep onset for >3 days/week for >6 months	Being pregnant or pregnancy (including given birth) in the preceding year
Not starting new treatments or medication and continuing their usual care 6 weeks prior to and during study participation (to obtain steady state)	Thoracic pain in absence of neck or low back pain
Refraining from analgesics, caffeine, alcohol, or nicotine in the previous 48h of the assessments	History of specific spinal surgery (e.g., surgery for spinal stenosis)
Nonspecific failed back surgery >3 years are permitted	Body Mass Index over 30
Not undertaking exercises (>3 MET) in the 3 days before the assessment	Presence of a current clinical depression diagnosed by a doctor

Table 1. In- and exclusion criteria

<u>Details polysomnography for eligibility screening</u>
An overnight home polysomnography (PSG) measurement using the Alice PDX portable sleep diagnostic system (Philips Respironics, Murrysville, PA, USA) was completed to verify study eligibility with regard to the exclusion of specific sleep disorders[1]. Obstructive sleep apnea was defined when the apnea-hypopnea index was over 15 and periodic leg movement disorder was defined as a periodic limb movement index over 15. The scoring of these indices followed the American Academy of Sleep Medicine Manual for Scoring of Sleep and Associated events guidelines[2].

Recruitment strategy:
Adults with nCSP and comorbid insomnia are recruited via flyers in the participating Universities and University Hospitals, primary care practices, occupational health services, public places, via advertisements and social media.

Sample size calculation:
Sample size (n=120) is calculated using G*Power based on the effects on pain in a pilot study (Cohen's f = 0.25, alpha=.05, power=.80),[3] accounted for F-tests and 20% loss to follow-up after one year.[4]

Randomisation:
Randomization is computer-generated at the Biostatistics Unit of Ghent University by an independent investigator, uninvolved in any other trial aspects. Block randomisation was used (ration 1:1) for the two treatment centres (Ghent; Brussels) separately, with stratification for sex (male; female) and dominant pain location (neck; low back).[13] The randomisation is performed by an independent researcher, who places papers indicating group assignment in sequentially numbered, opaque, and sealed envelopes. Concealed allocation is ensured since the independent researcher – who will be the only person with access to the envelops – writes the initials of the selected participants on the appropriate envelop before opening them.

Outcome measures:
<u>*Primary outcome:*</u>
The primary clinical outcome is defined as average pain intensity, assessed using the Brief Pain Inventory (BPI)[5] item 'average pain intensity in the last 24h' evaluated on an 11-point NRS (30% decrease is clinically important).[6]
<u>*Explorative, secondary pain-related outcomes:*</u>

- BPI worst pain intensity (last 24h) on an 11-point (0–10) numeric rating scale[5]
- BPI least pain intensity (last 24h) on an 11-point (0–10) numeric rating scale[5]
- BPI pain intensity now on an 11-point (0–10) numeric rating scale[5]
- BPI pain severity[5]: the mean of the four pain intensity items[5]
- BPI pain interference[5]: the BPI also evaluates the pain interference related to general activity, walking, work, mood, enjoyment of life, relation with others and sleep on separate 11-point numeric rating scales.
- The Central Sensitisation Inventory (CSI; cut-off value of 40/100 to indicate the presence of central sensitization-related symptoms) to assess symptoms of central sensitisation. The CSI uses 25 statements related to current health symptoms, indicative of central sensitization (scored on a five-point Likert scale ranging from zero to four).[7,8]
- Pressure pain thresholds (PPT; increase of >15% is clinically important[9]), defined as the point of minimum pressure that induces an unpleasant sensation is assessed with a digital pressure algometer (Wagner instruments), randomly applied at the painful and two remote locations.[10,11] The painful level is the trapezius muscle in neck pain patients and at L3 lumbar paravertebral muscles in low back pain patients. Remote sites are the web between the thumb and the index and the proximal one third of the calf. The threshold is determined as the mean of two consecutive measurements, with 30 s in between.

<u>*Explorative, secondary sleep-related outcomes:*</u>

- The Pittsburgh Sleep Quality Index (cut-off = 6/21; 3 points difference as clinically important[12,13,14]) to assess self-reported perceived sleep quality. It is a 19 item scale with 1 week time interval. Its score ranges between 0 and 21 and 0 indicates no sleep difficulty and 21 indicating severe sleep difficulties.
- The Insomnia Severity Index (cut-off = 14/28; 6 points difference as clinically important[13,15,16]) to assess self-reported insomnia severity. It is a 5-item 5-point Likert scale applying a 2 week time interval. Its scores ranges between 0 and 28 and 0 indicates no clinically significant insomnia and 28 indicating severe clinical insomnia.
- Dysfunctional Beliefs and Attitudes about Sleep[17,18] questionnaire to assess self-reported dysfunctional beliefs and attitudes about sleep. It is a 16-item 5-point Likert scale with scores ranging between 0 and 10. Scores over 4 indicate unrealistic expectations for sleep or unrealistic thoughts about sleep.
- The Epworth Sleepiness Scale[19] to assess self-reported sleep propensity. It is an 8-item 4-point Likert scale, with scores ranging between 0 and 24 and with 0 indicating normal daytime sleepiness and 24 indicating severe excessive daytime sleepiness.
- The Brugmann Fatigue Scale[20] to assess self-reported fatigue severity with mental fatigue subscale and physical fatigue subscale. The score ranges from 0 to 24 and higher scores indicate a higher subjective level of fatigue.

- Objective sleep outcomes derived from one night polysomnography assessment (portable Alice PDX, Philips Respironics IncTM). Participants were asked to refrain from caffeine after lunch, smoking in the evening and alcohol as well as intensive sport for the whole day. Participants were furthermore asked to take their regular medications and not to initiate new pharmacological treatments on the day of the polysomnography. Objective sleep quantity and architecture outcomes derived from the polysomnography recording were, at each time point, time in bed, total sleep time, sleep onset latency, wake duration after sleep onset, and early morning awakening in minutes; Non-Rapid Eye Movement (nREM) and REM sleep in % of total sleep time; Sleep efficiency in %; and number of arousals. The scoring of these sleep parameters followed the American Academy of Sleep Medicine Manual for Scoring of Sleep and Associated events guidelines[2]. The researcher who scored the polysomnography recording was blinded to group allocation.

Explorative, secondary other outcomes:

- The Hospital Anxiety and Depression rating scale (HADS)[21] to assess self-reported affective symptoms. It is a 14-item scale with depression subscale and anxiety subscale. Score ranges from 0 to 21, with 0 indicating absence of depression or anxiety and 21 indicating the presence of depression or anxiety. It includes a 7-item 4-point Likert 'depressive symptoms' subscale (0-21) and 7-item 4-point Likert 'Anxiety' subscale (0-21). Both apply a 4 week time interval
- The Short Form Health Survey-36 (SF-36)[22,23] to assess physical and mental functioning. The SF-36 is widely used a generic measure of health-related quality of life and constitutes well characterised psychometric properties[22,23].
- Physical activity recorded over 7 consecutive days by three-axis wrist worn accelerometers (GT9X-BT, Actigraph) will be used to define number of steps and percentage in level of activity (sedentary, light, moderate, vigorous) will be used as measure of physical activity.
- Adverse events classified as serious (led to death, life-threatening, required hospitalization, prolonged hospitalization, led to prolonged or significant disability).

Interventions:
Both groups receive 18 sessions of approx. 30 minutes over 14 weeks. All sessions are delivered by physical therapists (Master of Science), and are one-on-one, individualized sessions (except for one group pain educational session of 1h) using principles of person-centred care and applying guidance towards self-management. The experimental intervention compromises 6 sessions of CBTi, integrated in 12 sessions of BEPM (i.e., CBTi-BEPM).[24] The control intervention compromises of 18 sessions of BEPM (BEPM-only). The treatment contrast lies therefore in the 6 CBTi sessions. CBTi includes sleep education, self-monitoring of sleep patterns, time-in-bed restriction, stimulus control, sleep hygiene, cognitive restructuring, and relaxation.[25] For details, see[24-26]. BEPM includes pain neuroscience education (PNE; 3 sessions) and cognition-targeted exercise therapy. In CBTi-BEPM 9 sessions of cognition-targeted exercise therapy are provided, while in the BEPM-only intervention 15 sessions of cognition-targeted exercise therapy are provided to ensure balanced treatment arms regarding therapy/therapist exposure time. For full details on BEPM, see.[27]

Practical organization Best Evidence Pain Management (BEPM)
Table 2 shows the detailed practical organization of the 14-week control intervention, consisting of three sessions of Pain Neuroscience Education (PNE) and 15 sessions of Cognition-Targeted Exercise Therapy (CTET).

Week 1	Pain Neuroscience Education – 3 sessions (1 group session of 1 hour, 1 online session of 30 minutes, 1 face-to-face session of 30 minutes)	
Week 2		
Week 3	CTET (face-to-face, 30 minutes)	CTET (face-to-face, 30 minutes)
Week 4	CTET (face-to-face, 30 minutes)	CTET (face-to-face, 30 minutes)
Week 5	CTET (face-to-face, 30 minutes)	CTET (face-to-face, 30 minutes)
Week 6	CTET (face-to-face, 30 minutes)	
Week 7	CTET (face-to-face, 30 minutes)	
Week 8	CTET (face-to-face, 30 minutes)	
Week 9	CTET (face-to-face, 30 minutes)	
Week 10	CTET (face-to-face, 30 minutes)	

Week 11	CTET (face-to-face, 30 minutes)
Week 12	CTET (face-to-face, 30 minutes)
Week 13	CTET (face-to-face, 30 minutes)
Week 14	CTET (face-to-face, 30 minutes)
CTET = Cognition-Targeted Exercise Therapy	

Table 2. Organization of the therapeutic sessions

Therapeutic alliance between the therapist and patient was checked twice during the intervention, using an online questionnaire. This questionnaire was filled out by both the therapist and the patient after week 2 and week 8 of the intervention. It is important to note that the results of this questionnaire were not considered as trial outcomes. Instead, they served solely as a feedback tool, enabling therapists to address any issues in the therapeutic alliance as they arose.

Content

a. Pain Neuroscience Education (PNE) (3 sessions)

i. *First session*

The first session of PNE was held as a group session of a maximum of 6 people, which lasted about 1 hour. PNE aims to reconceptualize the patients' pain beliefs, to increase the patients' knowledge of pain and to decrease its threat value. The content will be based on current knowledge of the neurophysiology of pain according to Wall and Melzack[28], 'Pijneducatie, een praktische handleiding voor de (para)medici' by Van Wilgen and Nijs[29], and 'Explain Pain' by Butler and Moseley[30], including following topics in laymen's terms: (1) the neuron, (2) the synapse, (3) descending nociceptive inhibition and facilitation, (4) peripheral sensitization, and (5) central sensitization.

Information was provided to the participants using a PowerPoint, of which an example can be found using the following link: https://paininmotion.be/storage/ app/media//materials/sem-PainPhysiologyEducationEnglish.pdf

During the presentation specific questions were asked to the participant (e.g., "Wat symptoms do you have, and how do you cope with them right now?", "What comes to mind when hearing the terms acute and chronic pain, does that ring a bell?", "Can you explain in your own words what the nervous system is?"). These questions had several aims: they ensure active participation of the participants, lower the barriers to ask questions, and allow the therapist to individualize the content to the group. By the end of the session the goal was that participants were convinced that pain is not the direct result of tissue damage, but rather linked to hypersensitivity of the peripheral and central nervous system.

The session was closed by giving practical instructions on the online module (i.e., the second PNE session) and by providing an information leaflet. This leaflet mirrored the precise content covered in the group session. The participants were directed to read the leaflet, make note of any questions that may arise, and ideally, share the leaflet with a significant other. This sharing aimed to ensure that participants could receive social support during the intervention.

In this session, the completed feared activity forms were collected. Participants had initially received these forms during the baseline assessment, with instructions to complete them before the first therapy session. This timing was intentional to ensure that the information provided during this initial session did not influence the completion of the documents. Further details regarding the content and utilization of the feared activities form can be found in the description of the third educational session.

After closing the session, the therapist sends an e-mail to each of the participants including the link and instruction for the online module.

ii. *Second session*

The online module consisted of a website-embedded slideshow. Each slide featured a single sentence accompanied by a relevant image (refer to figure 1 for an illustration). The overarching purpose of the complete slideshow was to challenge misconceptions and erroneous beliefs associated with pain. Consequently, the content presented in the online slideshow mirrored the material covered in the group session. The full online module can be found at https://sites.google.com/view/onlinemodule/online-module.

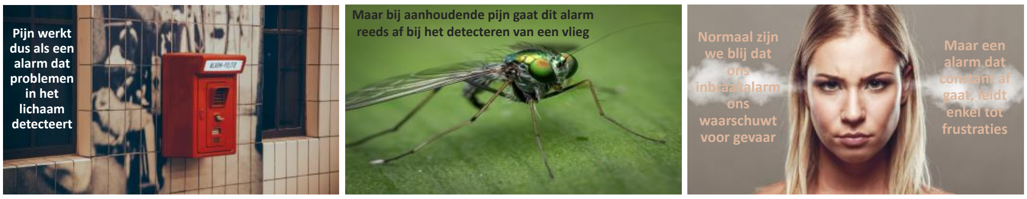

Figure 1. Example of slides used in the online slideshow.

Following the completion of the slideshow, the participants were asked to fill out a questionnaire. It's important to note that this questionnaire did not serve as an outcome measure in the trial; rather, it functioned exclusively as a practical tool for the therapist to tailor the content of the third educational session. The first part of this questionnaire featured the Neurophysiology of Pain test, designed to assess participants' understanding of the subject matter. This aspect helps identify specific areas that may require more in-depth discussion to ensure a comprehensive grasp of PNE. The second part of the questionnaire comprised open-ended questions to assess the participant's beliefs regarding their symptoms. Some examples are: "Do you believe that tissue damage is the root cause of your chronic pain issue?" "Do you think your nervous system is hypersensitive, and if so, what factors contribute to that?" "What actions can you take to exacerbate/alleviate symptoms?" and "Can your social environment play a role, and how does it cope with your chronic pain?".

iii. *Third session*

The third educational session was organized as an individual face-to-face session between the therapist and the participant. In preparation for this session, the therapist documented inaccuracies in the participant's responses to the Neurophysiology of Pain test and took note of significant comments or beliefs extracted from the open-ended questions. To further assist therapists, a session template was provided to guide the discussion in case no specific elements were evident from the questionnaire. Refer to table 3 for this template. It is essential to emphasize that not all aspects outlined in the template were obligatory for discussion, and the therapist had the flexibility to personalize the session by responding to the unique needs and narrative of the participant.

**SESSION 3**		
**LEAFLET AND ONLINE MODULE**	Questions and discussing provided information.	▪ After the first session, you received an information leaflet. Were you able to read it? Do you have any questions related to it? Did a significant other review it as well?<br>▪ Did everything go well with the online module? Do you have any questions related to its content?<br>▪ Reviewing responses to the questionnaire of the online module if needed. If relevant, focus on cause, personal explanation and maintaining factors of symptoms.
**UNDERSTANDING THE THERAPY CONCEPT**	Vicious circle 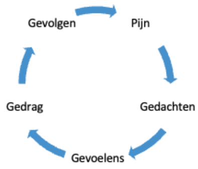	Try to use the participant's own words.<br>▪ What does this circle tell you?<br>▪ You tried to avoid activities and movements because you expected pain to alleviate, is that right? Do you feel that this strategy works for you and your symptoms?<br>▪ Sometimes, at short term, you felt pain alleviation, but in general the symptoms are still there and you seem not able to control them, right?<br>▪ Do you see any negative consequences of avoiding activities and movements?<br><br>***The aim of this circle is to conclude that inactivity leads to disuse and more pain. Avoidance can be beneficial at short term, but at long term this only maintains the pain problem.***<br><br>This circle also allows to explain the aims of the exercise program. We will try to break the circle by changing thoughts and behavior. Check with the participant if he/she understand the concept: "Do you have any idea why we will try to do that?".<br>"I want to propose that from now on, we will no longer focus on pain. Do you agree with that? Do you understand why I make this proposal? Do you have any idea why we cannot focus on pain anymore?" → This should lead to the conclusion that pain is no longer a reliable symptom, and that it cannot be used to shape therapy or impact daily life.
**THERAPEUTIC ALLIANCE**	Check if participant agrees with therapy concept	▪ I would like to engage in exercises with you, despite the discomfort you are experiencing. I aim to help your body recognize that you can perform these activities and movements, despite any fear or pain. Are you open to this? We will, of course, proceed gradually. If there is pain, we will no longer cease the activity, as we understand that pain is not a reliable indicator for you. Our goal is to retrain your body to understand that movement is not harmful. Does that resonate with you?<br>▪ Do you feel confident with this proposal? Do you understand why I am suggesting this approach to you?

Table 3. Template for third educational session

A crucial element of this third session involves reviewing the collaborative completion of the vicious circle with the participant. This facilitates discussion on the specific areas targeted for change in therapy, namely, thoughts and behavior. While PNE seeks to reshape thoughts and

beliefs about pain, the subsequent phase – CTET – aims to assist the participant in functional movement despite symptoms. This is achieved by progressively increasing activities and movements that hold significance for the participant.

b. Cognition-targeted exercise therapy (CTET) (15 sessions)
In the first session dedicated to CTET (i.e., the fourth session within the complete therapy structure) some time is allocated to setting both long-term and short-term goals. These goals are derived from the feared activities form that the participant provided to the therapist during the first educational session (refer to figure 2 for details of the feared activities form). This form asks the patient to list valued movements or activities they believe could exacerbate their complaints or disorder, and/or those that are restricted due to their (spinal) pain. The purpose of this form is to assist the therapist in identifying specific movements or activities that require attention during therapy. Collaboratively with the patients, these movements and activities are then categorized based on the level of fear or pain they evoke.

FEARED ACTIVITIES FORM

**Please document here the activities and/or movements you believe may exacerbate your symptoms and/or condition.**	**Level of certainty**<br>0------------------------------10<br>Not certain / Extremly certain
Lifting weights	10
Gardening and trimming the hedges	9
Remaining seated for a long time	7

Figure 2. Example of a filled out feared activities form by a chronic low back pain patient.

Goals were generally formulated SMART, and were always functional, never focusing on pain reduction or alleviation as pain is not a reliable symptom. If the patient wanted to focus on pain in the goals, the therapist asked questions on the content of the PNE sessions to allow the participant to understand that focusing on pain does not match the newly derived knowledge.

All exercise therapy sessions (n=15) were individual face-to-face sessions between the participant and the therapist, each lasting about 30 minutes. The therapists were free to choose specific exercises, if they fitted the gradual progression towards the functional goals and if they followed cognition-targeted principles[31]. These principles included[27]:

- All exercises are performed in a time-contingent manner ("Perform this exercise 10 times, regardless of the pain") rather than in a symptom-contingent way ("Stop or adjust the exercise when it hurts").
- Valued goal setting is done together with the patient, and the goals are focused on functionality instead of pain relief.
- The treating therapist continuously questions and challenges the patients' pain cognitions and perceptions and the expected outcome of each exercise, to change unhelpful cognitions and perceptions into positive ones. Instructions on the applied communication techniques are described extensively elsewhere[32].
- Exercises progress towards more feared movements and activities, and fears and unhelpful perceptions on negative consequences are discussed in this regard.
- Progression to more feared exercises is preceded by a phase of motor imagery, in patients with a high fear-level.

The purpose of CTET is to confront the patient with movements and activities that are feared, avoided and/or painful[27]. It was essential, both in physiotherapy sessions, during home exercises, and in daily life,

to steer clear of all 'safety behaviours' and emphasize normal and functional movements in a relaxed manner (i.e., without consciously contracting specific muscles or employing maladaptive compensation strategies). The progression aimed at advancing to more complex and more feared movements and activities. This progression is illustrated below, using the example of bending forward in a person suffering from chronic low back pain:

- Flexion in supine position - moving the knees towards the chest
- Flexion while being on hands and knees - moving the buttocks towards the heels
- Flexion while seated:
  - Reaching forward on a table while bending the back
  - Sustained flexion of the back while reaching left/right
- Flexion while standing:
  - Flexion of the back and returning to an upright position supported by one hand on a table
  - Flexion of the back without support
  - Sustained flexion of the back, while performing a rotation or side bending
  - Flexion of the back and returning to an upright position combined with rotation
- General principles for progression in each exercise:
  - Using increasing weights
  - Increasing the load arm of the used weights
  - Increasing the speed of exercises
  - Combining movements (e.g. flexion + rotation)
  - Progressing to an unstable base
  - Change in context/environment to increase threating value

The example aligns with what is typically observed in these patients. Naturally, this progression was tailored to each individual patient based on their specific feared movements. Similar principles were applied when introducing other movements or activities in both chronic low back and neck pain cases. Typically, the initial exercises mirrored the movement intended for the final activity but without triggering an association with the actual feared or painful movement. Ideally, these initial exercises induced some level of fear or stress to promote a successful learning experience leading to increased confidence.

Again, it should be acknowledged that communication on cognitions, perceptions and expectations was an essential component of cognition-targeted exercises as described above. The exercises were not used to target local aspects of the neck/back but were rather behavioural experiments targeting the brain. Specific examples of cognition-targeted communication during exercises between the physiotherapist and a chronic pain patient are extensively described elsewhere[32].

Several exercises, movements and activities used in the exercise program were also practiced at home. Therapists were instructed to use shared-decision principles to decide upon number, intensity and frequency of exercises, considering what exercises had been performed under the supervision of the therapist and what the participant considered feasible. It is crucial for the home exercises to be sufficiently challenging, yet the patient should feel confident to perform them independently at home. All home exercises were assigned with a focus on functionality rather than pure analytical movements and were applied using a time-contingent approach[27]. Moreover, communication on home exercises was equally important as communication during the exercises in therapy. At the start of each cognition-targeted exercise session, the last session and the home exercises were discussed, allowing to check therapy adherence, but also to check for potential problems or deterioration into old ‘biomechanical’ beliefs considering the pain problem. Upon occurrence, unhelpful beliefs were tackled by referring to the PNE sessions and by questioning the doubts of the participant.

During the very last session, the participants were provided with a document including the general principles of a cognition-targeted time-contingent exercise approach, as well as some examples of exercises that were performed during the intervention to ensure that they were able to maintain active after the end of therapy.

Practical organization Cognitive Behavioral Therapy for Insomnia integrated in Best Evidence Pain Management (CBTi-BEPM)

Table 4 shows the detailed practical organization of the 14-week experimental intervention, consisting of three sessions of Pain Neuroscience Education (PNE), nine sessions of Cognition-Targeted Exercise Therapy (CTET) and six sessions of Cognitive Behavioral Therapy for Insomnia (CBTi).

<table>
<tr><td>Week 1</td><td colspan="2" rowspan="2">Pain Neuroscience Education – 3 sessions
(1 group session of 1 hour, 1 online session of 30 minutes, 1 face-to-face session of 30 minutes)</td></tr>
<tr><td>Week 2</td></tr>
<tr><td>Week 3</td><td>CBTi (face-to-face, 30 minutes)</td><td>CTET (face-to-face, 30 minutes)</td></tr>
<tr><td>Week 4</td><td>CBTi (face-to-face, 30 minutes)</td><td>CTET (face-to-face, 30 minutes)</td></tr>
<tr><td>Week 5</td><td>CBTi (face-to-face, 30 minutes)</td><td>CTET (face-to-face, 30 minutes)</td></tr>
<tr><td>Week 6</td><td colspan="2">CBTi (face-to-face, 30 minutes)</td></tr>
<tr><td>Week 7</td><td colspan="2">CTET (face-to-face, 30 minutes)</td></tr>
<tr><td>Week 8</td><td colspan="2">CBTi (face-to-face, 30 minutes)</td></tr>
<tr><td>Week 9</td><td colspan="2">CTET (face-to-face, 30 minutes)</td></tr>
<tr><td>Week 10</td><td colspan="2">CBTi (face-to-face, 30 minutes)</td></tr>
<tr><td>Week 11</td><td colspan="2">CTET (face-to-face, 30 minutes)</td></tr>
<tr><td>Week 12</td><td colspan="2">CTET (face-to-face, 30 minutes)</td></tr>
<tr><td>Week 13</td><td colspan="2">CTET (face-to-face, 30 minutes)</td></tr>
<tr><td>Week 14</td><td colspan="2">CTET (face-to-face, 30 minutes)</td></tr>
<tr><td colspan="3">CBTi = Cognitive Behavioral Therapy for insomnia; CTET = Cognition-Targeted Exercise Therapy</td></tr>
</table>

Table 4. Organization of the therapeutic sessions

Therapeutic alliance between the therapist and patient was checked twice during the intervention, using an online questionnaire. This questionnaire was filled out by both the therapist and the patient after week 2 and week 8 of the intervention. It is important to note that the results of this questionnaire were not considered as trial outcomes. Instead, they served solely as a feedback tool, enabling therapists to address any issues in the therapeutic alliance as they arose.

Content

a. Pain Neuroscience education (PNE) (3 sessions)
   This part is identical in both content and practical organization as described above for the control group.

b. Cognition-targeted exercise therapy (CTET) (9 sessions)
   This part is identical in content as described above for the control group.
   However, where in the control group 15 sessions are dedicated to cognition-targeted exercise therapy, in the experimental group this part is limited to 9 sessions, to allow the other 6 sessions for cognitive behavioral therapy for insomnia (see description below).

c. Cognitive behavioral therapy for insomnia (CBTi) (6 sessions)
   All sessions dedicated to CBTi are collaboratively designed with the participants to align with their individual needs. Based on these needs, priorities for the intervention are determined, influencing whether to commence with one part or another (as outlined below). However, by the conclusion of the intervention, all components must be addressed by the therapist.

i. Sleep education

Sleep education was used to provide correct knowledge on sleep mechanisms and the development of chronic insomnia. This education (duration: approx. 30 minutes) was delivered using a PowerPoint and entails the fundament of the tips and skills learned during cognitive behavioral therapy for insomnia. Specific topic covered were: normal sleep and its phases, influence of age on sleep, what is insomnia?, development of insomnia with predisposing, provoking, and sustaining factors, the vicious circle of insomnia, and most important principles of cognitive behavioral therapy as an approach for tackling insomnia.

ii. Behavioral therapy

The initial phase of the behavioral therapy segment of the intervention involved establishing goals. This entailed a comparison of the current sleep schedule (incorporating factors such as sleep onset latency, number of nocturnal arousals, time spent in bed, and actual sleep duration) with the desired sleep schedule. The primary instrument in the behavioral aspect of CBTi is the sleep diary. Participants were required to maintain this diary throughout the entire intervention, serving both as a tracking tool and a means to assess therapy adherence. A manual on how to complete the sleep diary (see figure 3 for an example) was provided to participants, and the therapist also verbally explained the instructions during the initial use.

SLAAPDAGBOEK

DATUM	UREN																							
Week van ... tot	*			**																				
Maandag* - Dinsdag**	20	21	22	23	24	1	2	3	4	5	6	7	8	9	10	11	12	13	14	15	16	17	18	19
	Slaapkwaliteit: .../10				Waakkwaliteit: .../10				Gevoel overdag: .../10				Behandeling en opmerkingen:											
Dinsdag* - Woensdag**	20	21	22	23	24	1	2	3	4	5	6	7	8	9	10	11	12	13	14	15	16	17	18	19
	Slaapkwaliteit: .../10				Waakkwaliteit: .../10				Gevoel overdag: .../10				Behandeling en opmerkingen:											
Woensdag* - Donderdag**	20	21	22	23	24	1	2	3	4	5	6	7	8	9	10	11	12	13	14	15	16	17	18	19
	Slaapkwaliteit: .../10				Waakkwaliteit: .../10				Gevoel overdag: .../10				Behandeling en opmerkingen:											
Donderdag* - Vrijdag**	20	21	22	23	24	1	2	3	4	5	6	7	8	9	10	11	12	13	14	15	16	17	18	19
	Slaapkwaliteit: .../10				Waakkwaliteit: .../10				Gevoel overdag: .../10				Behandeling en opmerkingen:											
Vrijdag* - Zaterdag**	20	21	22	23	24	1	2	3	4	5	6	7	8	9	10	11	12	13	14	15	16	17	18	19
	Slaapkwaliteit: .../10				Waakkwaliteit: .../10				Gevoel overdag: .../10				Behandeling en opmerkingen:											
Zaterdag* - Zondag**	20	21	22	23	24	1	2	3	4	5	6	7	8	9	10	11	12	13	14	15	16	17	18	19
	Slaapkwaliteit: .../10				Waakkwaliteit: .../10				Gevoel overdag: .../10				Behandeling en opmerkingen:											
Zondag* - Maandag**	20	21	22	23	24	1	2	3	4	5	6	7	8	9	10	11	12	13	14	15	16	17	18	19
	Slaapkwaliteit: .../10				Waakkwaliteit: .../10				Gevoel overdag: .../10				Behandeling en opmerkingen:											

🡇 Uur van naar bed gaan A: Alcohol C: Caffeïne F: Fysieke activiteit Slaap of dutje R R R "Halve" slaap S S S Slaperigheid
🡅 Uur van opstaan M: Medicatie R: Roken Wakker V V V Vermoeidheid

Figure 3. Example of a blank sleep diary.

In short, the sleep diary enabled participants to visualize their bedtime, wake-up time, and any nocturnal awakenings. The use of colored squares visualizes periods of sleep, with blank squares indicating wakefulness. Additionally, participants can use the diary to monitor factors that may impact sleep, such as alcohol consumption, medication, caffeine intake, smoking, and physical activity. The diary also includes sections to track the perceived quality of sleep and daytime well-being. The following instructions regarding the use of the sleep diary were provided[33]:

- Complete the items from the previous night in the sleep diary about 20 to 30 minutes after waking up:
    - When you turn off the light to go the sleep, put a mark (downward arrow) to indicate the time of lights off.
    - When you turn on the light to get up in the morning, put a mark (upward arrow) to indicate the time of lights on.
    - When you read a book, watch television or do PC work in bed before light of, indicate this with a vertical line
    - Color the 15-minute boxes that you slept during the night
    - Outline other sleep disturbing factors
    - Rate your sleep quality
- Complete the daytime items in the sleep diary in the evening:
    - Use of alcohol, caffeine, medication (plus details), nicotine
    - Indicate when you took a nap.
    - Mark the times you felt sleepy or fatigued.
    - Rate your awakening quality and your mood
- General instructions:
    - Place your sleep diary in a visible location to enhance the likelihood of consistently filling it out.
    - Avoid clock monitoring during the night. The sleep diary of an overview of the perceived sleep.

From the sleep diary, sleep efficiency was computed as the ratio of total sleeping time to the total time spent in bed, serving as an indicator for sleep restriction therapy. Sleep efficiency represents a possible mismatch between time spent in bed and total sleep time. This therapy involves limiting the hours spent in bed as closely as possible to the actual hours of sleep (i.e. determining the sleep window). To achieve this, the time in bed was restricted to the mean sleep time of the previous week, as recorded in the sleep diary, which heightened sleep pressure. Consequently, excessive time in bed was addressed, and a mild form of sleep deprivation ensued. Importantly, the sleep window was never reduced below 5 hours per night, and the participant was not allowed to make any changes without consulting the therapist. In the long term, this approach facilitates faster sleep onset, improves sleep continuity, and results in a deeper, higher-quality sleep by stabilizing the circadian rhythm and balancing the production of melatonin and cortisol. Daytime naps were permitted, but their duration was restricted to a maximum of 15 minutes and only allowed if taken before 2 p.m.

Changes in the prescribed, restricted time in bed occurred using the following guidelines[33]:

- Sleep efficiency = 80-85% : sleep window is retained
- Sleep efficiency < 80% : a new sleep window is determined by making the total time in bed equal to the mean total sleep time of the past week (sleep window ≥5 hours because of safety and health concerns)
- Sleep efficiency > 85% : sleep window is extended by 15 minutes

Together with an efficacy > 85% any use of sleep-related medication was phased out in agreement with the participant and in close collaboration with the general practitioner of the participant.

For participants with high levels of anxiety, an exception was permitted. This allowed them to choose their bedtime freely one night per week to avoid heightened alertness caused by stress arising from sleep restriction therapy.

iii. Stimulus control

Insomniacs often develop habits that negatively impact their sleep patterns. For instance, falling asleep easily on the sofa during a favorite TV show but experiencing wakefulness when moving to bed afterward creates a conditioned association between the bedroom and wakefulness. Stimulus control aims to disrupt these conditioning patterns by reestablishing the connection between the bed/bedroom and sleep. The bedroom should be exclusively for sleeping and intimacy, while other activities like working, watching TV, and reading should occur in a different room. Participants were also encouraged to allocate an hour before bedtime for relaxing activities, linking relaxation with the bedroom and promoting healthy sleep conditioning. Additionally, participants were advised to only go to bed when feeling sleepy to enhance the likelihood of a swift onset of sleep and reduce the chances of rumination and worry. Specific instructions provided to participants included[33]:

- Retire to bed only when you feel sleepy.
- If unable to fall asleep within 15 minutes, rise from bed.
- Reserve your bed solely for sleep and intimacy.
- Avoid daytime napping.
- Maintain a consistent sleep-wake schedule.

iv. Sleep hygiene

Sleep hygiene covers advice on lifestyle (e.g., physical activity, diet, substance use) and environmental factors (e.g., temperature, noise, light). Though not typically the primary cause of chronic insomnia, these factors may contribute to ongoing sleep issues. Educating patients about these factors' relative risks aids treatment progress. The goal of sleep hygiene instructions was to address unfavorable sleep behaviors by providing information to enhance sleep quality. Several sleep hygiene instructions are given to patients[33], some of which align with time-in-bed restriction and stimulus control components:

- Refrain from consuming caffeine 4 to 6 hours before bedtime.
- Avoid smoking before bedtime or during nighttime awakenings.
- Steer clear of alcohol consumption 4 to 6 hours before bedtime.
- Abstain from heavy meals before going to bed.
- Refrain from intense physical training 2 hours before bedtime.

- Ensure your bedroom is a comfortable environment.
- Avoid extreme temperatures in your bedroom.
- Keep your bedroom both quiet and dark.
- Minimize exposure to active screens 1 hour before bedtime.

Caffeine and nicotine from smoking stimulate the central nervous system, negatively impacting sleep. Alcohol, while initially inducing sleep, results in reduced duration and poorer quality due to withdrawal symptoms. Therefore, using these substances before bedtime was discouraged. Intensive exercise close to bedtime activates the autonomic nervous system, raises body temperature, and delays sleep onset. However, physical activity at other times of the day promotes sleep and reduces stress.

v. Relaxation therapy

(Hyper)arousal is recognized as a contributing factor to the onset and persistence of insomnia. In turn, disrupted sleep can diminish a person's ability to cope with daily stressors. Relaxation therapy aimed to diminish somatic and/or cognitive arousal and its associated autonomic stress response, indirectly improving both sleep quality and pain. Using an information leaflet, participants were encouraged to practice relaxation techniques before bedtime using a self-management approach. This information leaflet included background information on the link between stress and sleep problems, detailed description of different relaxation techniques (i.e. Jacobson's method, visualization, mindfulness) and a detailed description on how the practice and implement these techniques in daily life.

vi. Cognitive therapy

The cognitive therapy component of CBTi targeted unrealistic expectations regarding sleep needs and daily functioning, misattributions and misconceptions about the causes of insomnia, distorted perceptions of consequences, and erroneous beliefs about sleep-promoting practices. The primary tool for cognitive therapy involved visualizing and discussing the vicious circle of thoughts, behavior, and emotions related to sleep. For instance, a participant might believe that 8 hours of sleep is the optimal amount (thought), leading to anxiety or anger when unable to achieve this goal (emotion). This, in turn, results in counting hours left to sleep and worrying about daytime effects (behavior), fostering mental activity and reducing chances of falling asleep.
Participants were assigned a home exercise to identify thoughts related to going to bed, being awake at night, and daytime effects. Subsequently, the therapist challenged these thoughts and explored alternatives with the participant. Importantly, thoughts were not minimized. Three examples are provided in table 5.

Example 1.
Dysfunctional cognition: "I feel that my insomnia is the result of my age"
Underlying belief: "There is nothing I can do to fix this problem, as I cannot change my age."
Cognitive error: Wrong assumption, wrong identification of the cause.
Intervention/Alternative interpretation: "Do all people of your age encounter the same sleep problems?" – "Apart from some normal changes in sleep structure, not all people of older age suffer from sleep problems. There are always psychological and behavioral factors linked to chronic insomnia, which are actually factors we can control and alter.".

Example 2.
Dysfunctional cognition: "After a night of bad sleep, I am unable to function during the day."
Underlying belief: "Sleep deprivation is detrimental for daytime functioning."
Cognitive error: Exaggeration, generalization.
Intervention/Alternative interpretation: "Did you exclusively experience negative effects and moments on the day(s) following a night of poor sleep?" - "Do the negative effects consistently manifest with the same intensity?" - "Are there potentially other factors contributing to these effects?"

Example 3.
Dysfunctional cognition: "My partner falls asleep one minute after going to bed, I need to be able to do the same."
Underlying belief: "Everyone has to sleep identically."
Cognitive error: Generalization, absolute and universal thinking.

Intervention/Alternative interpretation: “Do all individuals share the same height and weight?" - "Within certain normal boundaries, there exists significant variability among individuals regarding sleep onset latency, number of awakenings, sleep quality, and duration." - "It is advisable to refrain from social comparisons, as there will always be someone who sleeps better than you."

Table 5. Examples of maladaptive attitudes and beliefs regarding sleep and their alternatives

vii. Evaluation and relapse prevention

During the final CBTi session, the therapy was evaluated, and relapse prevention strategies were discussed. The evaluation involved reviewing apriori defined sleep-related goals and assessing the progress made toward achieving them. The patient's achievements were highlighted to motivate them to continue applying the same sleep principles post-therapy.

Relapse prevention aimed to help participants distinguish between an occasional bad night (acute insomnia) and a genuine relapse (chronic insomnia). Discussions covered potential risk factors, situations, and the role of the social network in assisting participants. The sleep diary was emphasized as a self-management tool for ongoing monitoring and intervention when signs of relapse appeared. Additionally, participants were provided with an informational leaflet summarizing key aspects of CBTi, including sleep education, the sleep diary, scoring system, sleep restriction therapy, stimulus control, sleep hygiene, and cognitive therapy. This resource served as a reference for participants to revisit and reinforce certain aspects as needed.

Statistical analysis plan

1. **Data source:** Data are derived from nCSP patients with insomnia who participate in this randomised controlled trial. They were evaluated at baseline, at post-intervention and at 3, 6 and 12 months follow-up. The primary outcome is average pain intensity in the last 24h assessed with the BPI.
2. **Analysis objectives:** To investigate the effectiveness of CBTi-BEPM versus BEPM in terms of the above-mentioned primary outcome at 12 months follow-up, and pain-related, sleep related and other secondary exploratory outcomes.
3. **Population:** nCSP patients with insomnia
4. **Endpoints:** Change in outcome measures from baseline to each follow-up point
5. **Covariates:** Sensitivity analyses performed for the primary outcome and all exploratory outcomes by controlling for baseline levels of pain intensity for pain-related outcomes and baseline levels of insomnia severity for sleep-related outcomes. These baseline levels were added to the random-intercept fixed slope linear mixed model analysis as confounding factor.
6. **Handling of missing data:** Analyses are carried out using linear mixed models which can handle missing data.
7. **Statistical methodology:**
   Descriptive statistics were used to report the demographic variables and baseline characteristics of the participating nCSP patients per intervention group, with means (Standard Deviation [SD], range and Inter Quartile Range [IQR]) for continuous variables and frequency (%) for categorical variables. All analyses were by intention-to-treat, with all participants included in the analysis according to their randomly allocated treatment. All analyses were performed in SPSS 24.0. For average pain intensity (primary outcome) and for all explorative, secondary outcomes, the change between baseline and other time points was calculated: Δ1 = baseline (T0) – post-treatment (T1); Δ2 = T0 – 3-month follow-up (T2); Δ3 = T0 – 6-month follow-up (T3); and Δ4 = T0 – 12-month follow-up (T4). Differences in the change in average pain intensity at 12-month follow-up (primary outcome at primary endpoint) and at the other time points (immediately post-intervention, and at 3- and 6-month follow-up) were analysed using a random-intercept fixed slope linear mixed model (incl. LSD post-hoc analyses), which was applied using an unstructured covariance matrix. Linear mixed models are a likelihood-based estimation procedure whereby likely values for missing data are estimated from information contained in the observed data, resulting in non-biased estimates providing data are missing at random. The model included treatment, time, and treatment*time as fixed effects together with a random intercept for each patient. Model assumptions were evaluated visually using residual plots. Mean group differences (95% confidence interval) at the different time points and their p-values and effect sizes for the intervention comparisons are reported. Level of significance for the primary outcome was set at α .05. Effect sizes were calculated as Cohen’s d (interpreted as >1.3 = very large; .80-1.29 = large, .50-.79 = medium, .20-.49 = small, <.20

= negligible). The same analysis was used to evaluate the explorative, secondary outcomes at the different time points.
Sensitivity analyses were performed using the same analysis, including the baseline level of average pain intensity (for all pain-related outcomes) and the baseline level of insomnia severity (for sleep-related outcomes) as confounding factor. The analysis of dropout and loss-to-follow-up was conducted by categorizing the entire cohort into two subsets, distinguished by their adherence or discontinuation from the trial (which also included loss-to-follow-up). Comparative assessment of baseline characteristics and data between the two groups was accomplished utilizing a t-test or its non-parametric equivalent.

References

1. Nilius G, Domanski U, Schroeder M, et al. A randomized controlled trial to validate the Alice PDX ambulatory device. *Nature and science of sleep* 2017; **9**: 171-80.
2. Berry RB, Brooks R, Gamaldo CE, Harding SM, Marcus C, Vaughn BV. The AASM manual for the scoring of sleep and associated events. *Rules, Terminology and Technical Specifications, Darien, Illinois, American Academy of Sleep Medicine* 2012; **176**: 2012.
3. Jungquist CR, O'Brien C, Matteson-Rusby S, et al. The efficacy of cognitive-behavioral therapy for insomnia in patients with chronic pain. *Sleep medicine* 2010; **11**(3): 302-9.
4. Malfliet A, Kregel J, Coppieters I, et al. Effect of Pain Neuroscience Education Combined With Cognition-Targeted Motor Control Training on Chronic Spinal Pain: A Randomized Clinical Trial. *JAMA neurology* 2018; **75**(7): 808-17.
5. Stanhope J. Brief Pain Inventory review. *Occupational Medicine* 2016; **66**(6): 496-7.
6. Dworkin RH, Turk DC, Wyrwich KW, et al. Interpreting the clinical importance of treatment outcomes in chronic pain clinical trials: IMMPACT recommendations. *The journal of pain* 2008; **9**(2): 105-21.
7. Cuesta-Vargas AI, Neblett R, Chiarotto A, et al. Dimensionality and Reliability of the Central Sensitization Inventory in a Pooled Multicountry Sample. *The journal of pain* 2018; **19**(3): 317-29.
8. Kregel J, Vuijk PJ, Descheemaeker F, et al. The Dutch Central Sensitization Inventory (CSI): Factor Analysis, Discriminative Power, and Test-Retest Reliability. *The Clinical journal of pain* 2016; **32**(7).
9. Fischer A. Application of pressure algometry in manual medicine. *J Man Med* 1990; **5**(4): 145-50.
10. Chesterton LS, Sim J, Wright CC, Foster NE. Interrater Reliability of Algometry in Measuring Pressure Pain Thresholds in Healthy Humans, Using Multiple Raters. *The Clinical journal of pain* 2007; **23**(9): 760-6.
11. Cathcart S, Pritchard D. Reliability of pain threshold measurement in young adults. *The Journal of Headache and Pain* 2006; **7**(1): 21-6.
12. Buysse DJ, Reynolds CF, 3rd, Monk TH, Berman SR, Kupfer DJ. The Pittsburgh Sleep Quality Index: a new instrument for psychiatric practice and research. *Psychiatry research* 1989; **28**(2): 193-213.
13. Alsaadi SM, McAuley JH, Hush JM, et al. Detecting insomnia in patients with low back pain: accuracy of four self-report sleep measures. *BMC musculoskeletal disorders* 2013; **14**(1): 196.
14. Eadie J, van de Water AT, Lonsdale C, et al. Physiotherapy for sleep disturbance in people with chronic low back pain: results of a feasibility randomized controlled trial. *Arch Phys Med Rehabil* 2013; **94**(11): 2083-92.
15. Bastien CH, Vallieres A, Morin CM. Validation of the Insomnia Severity Index as an outcome measure for insomnia research. *Sleep medicine* 2001; **2**(4): 297-307.
16. Yang M, Morin CM, Schaefer K, Wallenstein GV. Interpreting score differences in the Insomnia Severity Index: using health-related outcomes to define the minimally important difference. *Current Medical Research and Opinion* 2009; **25**(10): 2487-94.
17. Blais FC, Gendron L, Mimeault V, Morin CM. [Evaluation of insomnia: validity of 3 questionnaires]. *L'Encephale* 1997; **23**(6): 447-53.
18. Morin CM, Vallieres A, Ivers H. Dysfunctional beliefs and attitudes about sleep (DBAS): validation of a brief version (DBAS-16). *Sleep* 2007; **30**(11): 1547-54.

19. Johns MW. A new method for measuring daytime sleepiness: the Epworth sleepiness scale. *Sleep* 1991; **14**(6): 540-5.
20. Mairesse O, Damen V, Newell J, Kornreich C, Verbanck P, Neu D. The Brugmann Fatigue Scale: An Analogue to the Epworth Sleepiness Scale to Measure Behavioral Rest Propensity. *Behavioral sleep medicine* 2019; **17**(4): 437-58.
21. LoMartire R, Äng BO, Gerdle B, Vixner L. Psychometric properties of Short Form-36 Health Survey, EuroQol 5-dimensions, and Hospital Anxiety and Depression Scale in patients with chronic pain. *Pain* 2020; **161**(1): 83-95.
22. Aaronson NK, Muller M, Cohen PD, et al. Translation, validation, and norming of the Dutch language version of the SF-36 Health Survey in community and chronic disease populations. *J Clin Epidemiol* 1998; **51**(11): 1055-68.
23. Ware J, Snow KK, Kosinski M, Gandek B. SF-36 Health Survey Manual and Interpretation Guide. Boston, MA, USA: The Health Institute, New England Medical Center; 1993.
24. Van Looveren E, Meeus M, Cagnie B, et al. Combining Cognitive Behavioral Therapy for Insomnia and Chronic Spinal Pain Within Physical Therapy: A Practical Guide for the Implementation of an Integrated Approach. *Phys Ther* 2022; **102**(8).
25. Perlis ML B-JC, Smith MT, Posner DA. . Cognitive behavioral treatment of insomnia: A session-by-session guide: Springer New York; 2005.
26. Nijs J, Mairesse O, Neu D, et al. Sleep Disturbances in Chronic Pain: Neurobiology, Assessment, and Treatment in Physical Therapist Practice. *Physical therapy* 2018.
27. Malfliet A, Kregel J, Meeus M, et al. Applying contemporary neuroscience in exercise interventions for chronic spinal pain: treatment protocol. *Brazilian journal of physical therapy* 2017; **21**.
28. Wall P, Melzack R. Textbook of Pain. 1999.
29. van Wilgen CP, Nijs J. Pijneducatie - een praktische handleiding voor (para)medici. 2010.
30. Butler DS, Moseley GL. Explain Pain. 2003.
31. Nijs J, Meeus M, Cagnie B, et al. A modern neuroscience approach to chronic spinal pain: combining pain neuroscience education with cognition-targeted motor control training. *Physical therapy* 2014; **94**: 730-8.
32. Nijs JJ, Lluch Girbes E, Lundberg M, et al. Exercise therapy for chronic musculoskeletal pain: Innovation by altering pain memories. *Manual therapy* 2015; **20**: 216-20.
33. Van Looveren E, Meeus M, Cagnie B, et al. Combining Cognitive Behavioral Therapy for Insomnia and Chronic Spinal Pain Within Physical Therapy: A Practical Guide for the Implementation of an Integrated Approach. *Physical Therapy* 2022; **102**(8).
