# Supplementary material for: Cognitive Behavioral Therapy for Insomnia in Pain Management for Nonspecific Chronic Spinal Pain: A Randomized Clinical Trial
Source: JAMA Netw Open. 2024 Aug 9;7(8):e2425856. doi: 10.1001/jamanetworkopen.2024.25856 (PMC11316234; doi:10.1001/jamanetworkopen.2024.25856)
Supplement: Supplement 2. — eTable 1. Inclusion and Exclusion Criteria eMethods. Details on Polysomnography Assessment eTable 2. Training and Supervision of Therapists eTable 3. Overview of Missing Data per Outcome and per Time Point eFigure 1. Forest Plot Showing the Results of the Analyses Using Delta Values for Self-Reported Pain-Related Outcomes eFigure 2. Forest Plot Showing the Results of the Analyses Using Delta Values for Self-Reported Sleep-Related Outcomes eTable 4. Sensitivity Analyses eFigure 3. Forest Plot Showing the Results of the First Sensitivity Analyses Using Delta Values for Self-Reported Pain-Related Outcomes eFigure 4. Forest Plot Showing the Results of the First Sensitivity Analyses Using Delta Values for Self-Reported Sleep-Related Outcomes eTable 5. Second Sensitivity Analyses, Comparing Dropouts to Non-dropouts eTable 6. Remitters, Responders and NNT Analyses eTable 7. Success of Assessor and Participant Blinding eTable 8. Absolute Values at Each Time Point eReferences [file jamanetwopen-e2425856-s002.pdf]

Supplementary Online Content

Malfliet A, De Baets L, Thomas Bilterys T, et al. Cognitive behavioral therapy for insomnia in pain management for nonspecific chronic spinal pain: a randomized clinical trial. *JAMA Netw Open*. 2024;7(8):e2425856. doi:10.1001/jamanetworkopen.2024.25856

eTable 1. Inclusion and Exclusion Criteria

eMethods. Details on Polysomnography Assessment

eTable 2. Training and Supervision of Therapists

eTable 3. Overview of Missing Data per Outcome and per Time Point

eFigure 1. Forest Plot Showing the Results of the Analyses Using Delta Values for Self-Reported Pain-Related Outcomes

eFigure 2. Forest Plot Showing the Results of the Analyses Using Delta Values for Self-Reported Sleep-Related Outcomes

eTable 4. Sensitivity Analyses

eFigure 3. Forest Plot Showing the Results of the First Sensitivity Analyses Using Delta Values for Self-Reported Pain-Related Outcomes

eFigure 4. Forest Plot Showing the Results of the First Sensitivity Analyses Using Delta Values for Self-Reported Sleep-Related Outcomes

eTable 5. Second Sensitivity Analyses, Comparing Dropouts to Non-dropouts

eTable 6. Remitters, Responders and NNT Analyses

eTable 7. Success of Assessor and Participant Blinding

eTable 8. Absolute Values at Each Timepoint

eReferences

This supplementary material has been provided by the authors to give readers additional information about their work.

eTable 1 – Inclusion- and exclusion criteria

Inclusion	Exclusion
Nonspecific spinal pain for at least 3 months' duration, at least 3 days/week	Severe underlying sleep pathology (identified through baseline data of polysomnography)
Aged between 18 and 65 years	Neuropathic pain
Seeking care because of neck pain or low back pain	Chronic widespread pain syndromes
Native Dutch Speaker	Shift workers
Having insomnia: in the absence of other intrinsic sleep-disorders and shift work, insomnia is defined as > 30 min of sleep latency and/or minutes awake after sleep onset for >3 days/week for >6 months	Being pregnant or pregnancy (including given birth) in the preceding year
Not starting new treatments or medication and continuing their usual care 6 weeks prior to and during study participation (to obtain steady state)	Thoracic pain in absence of neck or low back pain
Refraining from analgesics, caffeine, alcohol, or nicotine in the previous 48h of the assessments	History of specific spinal surgery (e.g., surgery for spinal stenosis)
Nonspecific failed back surgery >3 years are permitted	Body Mass Index over 30
Not undertaking exercises (>3 MET) in the 3 days before the assessment	Presence of a current clinical depression diagnosed by a doctor

© 2024 Malfliet A et al. *JAMA Network Open.*

eMethods. Details on polysomnography assessment

Details polysomnography during screening

An overnight home polysomnography (PSG) measurement using the Alice PDX portable sleep diagnostic system (Philips Respironics, Murrysville, PA, USA) was completed to verify study eligibility with regard to the exclusion of specific sleep disorders[1]. Obstructive sleep apnea was defined when the apnea-hypopnea index was over 15 and periodic leg movement disorder was defined as a periodic limb movement index over 15. The scoring of these indices followed the American Academy of Sleep Medicine Manual for Scoring of Sleep and Associated events guidelines[2].

Polysomnography for outcomes assessment

Overnight objective sleep data recorded via polysomnography were collected in the comfort of the participant's home during one night at baseline (same assessment as the screening polysomnography assessment), immediately post-intervention and at 12 months post-intervention. Participants were asked to refrain from caffeine after lunch, smoking in the evening and alcohol as well as intensive sport for the whole day. Participants were furthermore asked to take their regular medications and not to initiate new pharmacological treatments on the day of the polysomnography. Objective sleep quantity and architecture outcomes derived from the polysomnography recording were, at each time point, time in bed, total sleep time, sleep onset latency, wake duration after sleep onset, and early morning awakening in minutes; Non-Rapid Eye Movement (nREM) and REM sleep in % of total sleep time; Sleep efficiency in %; and number of arousals. The scoring of these sleep parameters followed the American Academy of Sleep Medicine Manual for Scoring of Sleep and Associated events guidelines[2]. The researcher who scored the polysomnography recording was blinded to group allocation.

eTable 2.Training and supervision of therapists

All therapy sessions were provided by experienced master-level physiotherapists specifically trained in the study interventions. To avoid treatment contamination, therapists are only trained in the intervention for their group and cannot switch groups after training. Control therapists are not allowed to have prior training in Cognitive Behavioral Therapy for insomnia. Participants were always treated by the same trial therapist during the complete intervention. All training was based on standardized intervention manuals and followed a standardized structure.

<table>
<tr><th>CBTi-BEPM Therapists</th><th>BEPM therapists</th></tr>
<tr><td>General face-to-face session (1,5h) containing:
1. Information on the study organization
2. Flow of the study participants
3. Timeline of the study and therapy
4. Principles of good clinical practice
5. Treatment manuals
6. Content and structure of the sessions
7. Demonstration of Pain Neuroscience Education (PNE) presentation

Training on BEPM included:
1. Individual preparation at home to perform PNE in a mock situation together with the trainers (approx. 2h).
2. Individual face-to-face session (3h) including mock PNE presentation with ad hoc feedback from the trainers. This session also covered the principles and content of cognition-targeted exercise therapy, with some case examples.
3. Individual preparation at home to master principles and content of CTET (approx. 2h).
4. Individual face-to-face session (2h) to cover CTET using case examples and an extensive Q&A.

Training on CBTi included:
1. 4-day course on CBTi delivered by the Belgian Association of Sleep research and Sleep Medicine (22h), covering theory on sleep and sleep problems, pharmacological interventions, theory and practical application of CBTi.
2. Two lectures on insomnia and CBTi (4h) at the faculty of Psychology of the Vrije Universiteit Brussel.
3. Observational internship (14h) during group CBTi sessions delivered by a certified somnologist for a group of individuals experiencing insomnia.</td><td>General face-to-face session (1,5h) containing:
1. Information on the study organization
2. Flow of the study participants
3. Timeline of the study and therapy
4. Principles of good clinical practice
5. Treatment manuals
6. Content and structure of the sessions
7. Demonstration of Pain Neuroscience Education (PNE) presentation

Training on BEPM included:
1. Individual preparation at home to perform PNE in a mock situation together with the trainers (approx. 2h).
2. Individual face-to-face session (3h) including mock PNE presentation with ad hoc feedback from the trainers. This session also covered the principles and content of cognition-targeted exercise therapy, with some case examples.
3. Individual preparation at home to master principles and content of CTET (approx. 2h).
4. Individual face-to-face session (2h) to cover CTET using case examples and an extensive Q&A.</td></tr>
</table>

Every quarter, a dedicated session was arranged for therapists (EXP and CON) and trainers to review and address any issues, to avoid any therapy drift, as well as to offer additional information or training as required. Moreover, in case of problems, doubts or any questions, the therapists were in close contact with the trainers through e-mail. If needed, additional online meetings were planned to tackle any issue.

© 2024 Malfliet A et al. *JAMA Network Open.*

Additionally, the therapists were instructed to use checklists specifically constructed for each therapy session to ensure that no aspects were forgotten. An example of such a checklist can be found in eFigure 1. Moreover, the specific content of each therapy session related to CTET or CBTi was captured by the therapists in a written lab notebook.

eTable 3. Overview of missing data per outcome and per time point

		Experimental intervention (n=61 included)				Control intervention (n=62 included)			
		Drop-out	Loss-to-follow-up	Variable missing data	**Total missing**	Drop-out	Loss-to-follow-up	Variable missing data	**Total missing**
PSG	T1	6	1	1	**8**	7	1	4	**12**
	T4	6	7	13	**26**	7	2	12	**21**
ACT	T1	6	1	4	**11**	7	1	6	**14**
	T4	6	7	10	**23**	7	2	15	**24**
PPT	T1	6	1	0	**7**	7	1	2	**10**
	T4	6	7	8	**21**	7	2	7	**16**
Q	T1	6	1	0	**7**	7	1	0	**8**
	T2	6	1	0	**7**	7	1	0	**8**
	T3	6	3	1	**9**	7	1	0	**8**
	T4	6	7	1	**14**	7	2	1	**10**

PSG: Polysomnography; ACT: Actigraphy; PPT: Pressure Pain Thresholds; Q: Questionnaires.
T1: immediately post-intervention; T2: 3 months post-intervention (questionnaires only); T3: 6 months post-intervention (questionnaires only); T4: 12 months post-intervention.

Table 7. Overview of total amount of missing data per outcome and time point

© 2024 Malfliet A et al. *JAMA Network Open.*

Visual representation of the results of the analyses using delta values by means of Forest Plots

Two Forest Plots are presented to visually show the results of the main analysis at post-intervention and 1 year follow-up, with regard to self-reported pain outcomes (primary outcome average pain intensity and secondary, exploratory pain-related outcomes – Figure 7) and secondary, exploratory self-reported sleep-outcomes (Figure 8).

© 2024 Malfliet A et al. *JAMA Network Open.*

eFigure 1. Forest Plot showing the results of the analyses using delta values for self-reported pain-related outcomes.

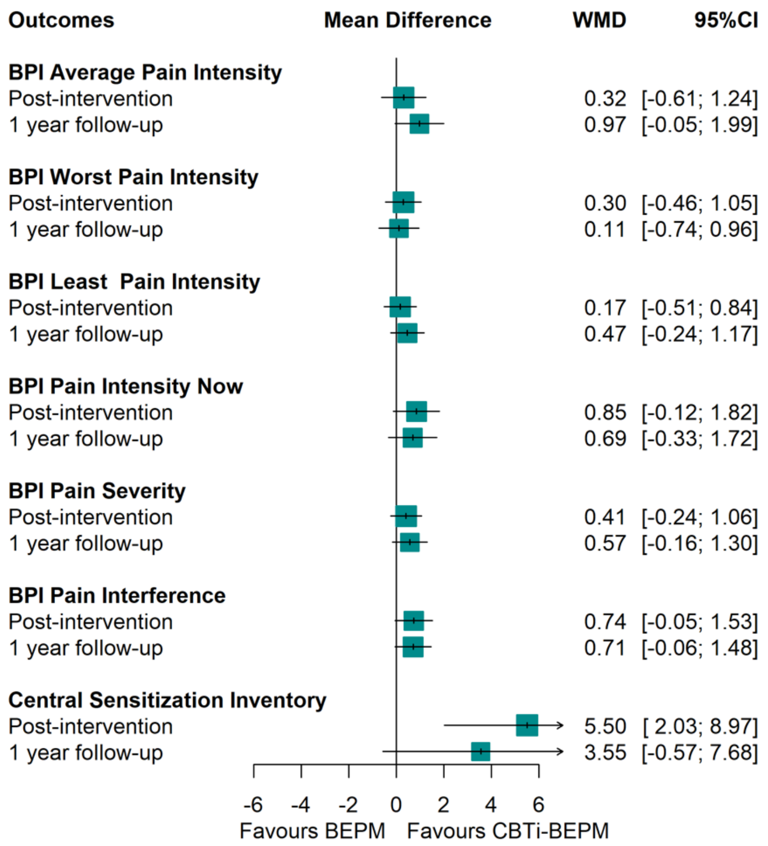

Legend. BPI: Brief Pain Inventory; WMD: Weighted Mean Differences; CBTi-BEPM: Cognitive Behavioral Therapy for Insomnia integrated in Best-Evidence Pain Management; BEPM: Best-Evidence Pain Management

© 2024 Malfliet A et al. *JAMA Network Open.*

eFigure 2. Forest Plot showing the results of the analyses using delta values for self-reported sleep-related outcomes.

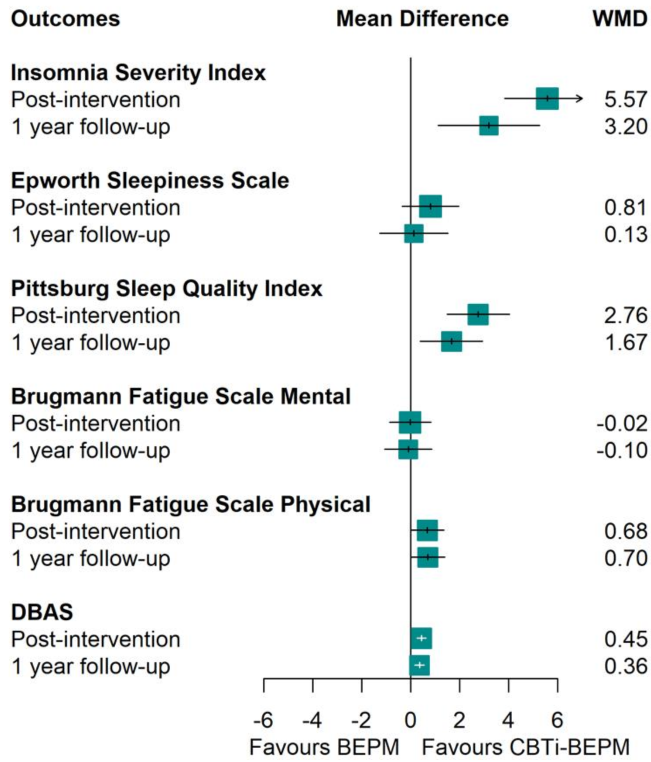

Legend. DBAS: Dysfunctional Beliefs About Sleep; WMD: Weighted Mean Differences; CBTi-BEPM: Cognitive Behavioral Therapy for Insomnia integrated in Best-Evidence Pain Management; BEPM: Best-Evidence Pain Management

© 2024 Malfliet A et al. *JAMA Network Open.*

eTable 4. Sensitivity Analyses

Different sensitivity analyses were performed. The first sensitivity analyses (presented in table 8 and Figure 9 and 10) were performed for the primary outcome and all exploratory outcomes by controlling for baseline levels of pain intensity for pain-related outcomes and baseline levels of insomnia severity for sleep-related outcomes. As such, these baseline levels were added to the random-intercept fixed slope linear mixed model analysis as confounding factor.
The second sensitivity analysis (presented in table 9) focused on comparing the dropout group, including participants loss-to-follow-up, with the no dropout group. This was conducted by categorizing the entire cohort into two subsets, distinguished by their adherence or discontinuation from the trial (which also included loss-to-follow-up). Comparative assessment of baseline characteristics and data between the two groups was accomplished utilizing a t-test or its non-parametric equivalent.

		CBTi-BEPM (n=61)	BEPM (n=62)	Mean group difference [95% CI]	p-value
Primary outcome – Average Pain Intensity†					
**Brief Pain Inventory Average Pain Intensity** (0-10)	Δ1	1.485±.301	1.600±.299	-.115 [.726;-.956]	p=.787
	Δ2	1.127±.314	1.363±.312	-.235 [.643;-1.114]	p=.597
	Δ3	1.545±.276	1.590±.270	-.0386 [.720;-.811]	p=.907
	Δ4	1.679±.318	1.233±.310	.444 [1.327;-.434]	p=.317
Secondary pain related outcomes†					
**Brief Pain Inventory Worst Pain Intensity** (0-10)	Δ1	1.207±.275	.984±.273	.224 [.991;-.544]	p=.565
	Δ2	.883±.310	1.144±.308	-.206 [.607;-1.128]	p=.553
	Δ3	1.364±.317	1.134±.311	.230 [1.110;-.651]	p=.606
	Δ4	1.034±.313	.978±.303	.055 [.920;-.810]	p=.899
**Brief Pain Inventory Least Pain Intensity** (0-10)	Δ1	.821±.238	.845±.236	-.025 [.640;-.689]	p=.941
	Δ2	.929±.227	1.197±.225	-.268 [.366;-.902]	p=.404
	Δ3	.914±.267	.822±.263	.092 [.834;-.650]	p=.806
	Δ4	1.301±.253	1.020±.248	.281 [.983;-.421]	p=.429
**Brief Pain Inventory Pain Intensity Now** (0-10)	Δ1	1.570±.334	1.054±.332	.517 [1.450;-.417]	p=.275
	Δ2	1.050±.326	1.446±.324	-.396 [.515;-1.308]	p=.391
	Δ3	1.495±.304	1.153±.298	.343 [-1.187;-.502]	p=.423
	Δ4	1.399±.329	1.196±.319	.203 [1.113;-.707]	p=.659
**Brief Pain Inventory Pain Severity** (0-10)	Δ1	1.271±.218	1.121±.217	.150 [.760;-.460]	p=.627
	Δ2	.997±.233	1.287±.232	-.290 [.361;-.941]	p=.379
	Δ3	1.325±.223	1.175±.219	.151 [.771;-.470]	p=.631
	Δ4	1.352±.241	1.104±.235	.248 [.915;-.419]	p=.462
**Brief Pain Inventory Pain Interference** (0-10)	Δ1	1.539±.273	1.064±.271	.475 [1.239;-.289]	p=.220
	Δ2	1.642±.259	1.087±.258	.555 [1.280;-.170]	p=.132
	Δ3	1.675±.275	1.089±.270	.586 [1.351;-.179]	p=.132
	Δ4	1.630±.262	1.220±.256	.411 [1.137;-.316]	p=.265
**Central Sensitization Inventory** (0-100)	Δ1	12.341±1.242	7.429±1.235	4.912 [8.385;1.439]	**p=.006**
	Δ2	10.170±1.356	8.837±1.348	1.334 [5.126;-2.459]	p=.487
	Δ3	10.632±1.514	6.309±1.492	4.323 [8.542;.104]	**p=.045**
	Δ4	10.413±1.500	7.440±1.467	2.973 [7.136;-1.189]	p=.160
**Pressure Pain Thresholds Primary*** (kgf)	Δ1	-1.254±.236	-.638±.241	.616 [.059;-1.291]	p=.073
	Δ4	-1.109±.292	-1.165±.279	-.056 [.862;-.750]	p=.891
**Pressure Pain Thresholds Calf Secondary** (kgf)	Δ1	-.507±.188	-.338±.189	-.169 [.359;-.697]	p=.528
	Δ4	-.826±.258	-.805±.247	-.021 [.687;-.730]	p=.953
**Pressure Pain Thresholds Hand Secondary** (kgf)	Δ1	-.601±.147	-.183±.148	-.418 [-.004;-.832]	**p=.048**
	Δ4	-.861±.193	-.725±.181	-.136 [.390;-.661]	p=.609
Secondary sleep related outcomes‡					
**Insomnia Severity Index**	Δ1	7.777±.597	3.189±.588	4.588 [6.250;-2.927]	**p<.001**
	Δ2	7.310±.658	4.249±.649	3.061 [4.893;1.230]	**p=.001**

© 2024 Malfliet A et al. *JAMA Network Open.*

(0-28)	Δ3	7.417±.707	3.863±.692	5.554 [5.516;1.591]	**p<.001**
	Δ4	6.695±.732	4.474±.709	2.222 [4.244;.200]	**p=.032**
**Epworth Sleepiness Scale** (0-24)	Δ1	1.224±.431	.439±.425	.785 [1.986;-.415]	p=.198
	Δ2	1.537±.534	1.166±.527	.370 [1.858;-1.117]	p=.622
	Δ3	1.735±.546	1.134±.535	.601 [2.118;-.916]	p=.434
	Δ4	1.527±.522	1.277±.507	.250 [1.695;-1.195]	p=.732
**Pittsburg Sleep Quality Index** (0-21)	Δ1	4.361±.465	2.002±.459	2.359 [3.654;1.064]	**p<.001**
	Δ2	4.280±.414	2.717±.408	1.562 [2.715;.410]	**p=.008**
	Δ3	4.050±.475	2.392±.464	1.659 [2.976;.341]	**p=.014**
	Δ4	3.781±.472	2.405±.458	1.377 [2.683;.071]	**p=.039**
**Brugmann Fatigue Scale Mental** (0-12)	Δ1	.729±.308	1.024±.304	-.295 [.563;-1.152]	p=.497
	Δ2	.968±.334	1.312±.329	-.344 [.586;-1.274]	p=.465
	Δ3	1.023±.363	.988±.355	.036 [1.044;-.972]	p=.944
	Δ4	.760±.357	1.063±.349	-.303 [.688;-1.294]	p=.546
**Brugmann Fatigue Scale Physical** (0-12)	Δ1	.834±.243	.446±.239	.388 [1.064;-.287]	p=.257
	Δ2	1.152±.272	.494±.268	.658 [1.415;-.099]	p=.088
	Δ3	1.131±.299	.202±.293	.929 [1.759;.099]	**p=.029**
	Δ4	1.243±.258	.618±.251	.625 [1.339;-.089]	p=.086
**DBAS** (0-10)	Δ1	.693±.070	.273±.069	.419 [.614;.225]	**p<.001**
	Δ2	.705±.073	.335±.072	.370 [.574;.167]	**p<.001**
	Δ3	.767±.073	.390±.072	.378 [.580;.175]	**p<.001**
	Δ4	.740±.071	.419±.069	.321 [.517;.126]	**p=.002**
**PSG – Sleep Onset Latency** (min)	Δ1	1.370±1.907	3.433±1.933	-2.063 [3.323;-7.450]	p=.449
	Δ4	1.928±3.697	-4.557±3.528	6.485 [16.647;-3.676]	p=.208
**PSG Wake After Sleep Onset** (min)	Δ1	3.224±5.562	14.403±5.595	-11.179 [4.472;-26.830]	p=.160
	Δ4	-2.227±7.560	4.240±7.560	-6.468 [14.272;-27.208]	p=.536
**PSG Early Morning Awakenings** (min)	Δ1	-.215±1.955	-4.012±1.971	3.797 [9.306;-1.711]	p=.174
	Δ4	-2.275±3.664	-8.855±3.458	6.579 [16.617;-3.459]	p=.196
**PSG Time In Bed** (min)	Δ1	5.978±11.487	12.997±11.589	-7.320 [25.043;-39.682]	p=.655
	Δ4	-15.970±13.308	.116±12.843	-16.086 [20.642;-52.814]	p=.387
**PSG Total Sleep Time** (min)	Δ1	5.445±11.042	4.734±11.045	.711 [31.691;-30.269]	p=.964
	Δ4	-8.096±13.145	15.786±12.587	-23.883 [12.280;-60.045]	p=.193
**PSG Sleep Efficiency** (%)	Δ1	.266±1.289	-1.176±1.294	1.442 [5.066;-2.181]	p=.432
	Δ4	1.559±1.636	3.047±1.555	-1.488 [3.004;-5.981]	p=.512
**PSG REM-sleep** (%)	Δ1	-4.717±1.126	-5.324±1.129	.607 [3.770;-2.556]	p=.704
	Δ4	-3.635±1.263	-5.398±1.203	1.763 [5.239;-1.712]	p=.315
**PSG non-REM-sleep** (%)	Δ1	4.707±1.127	5.314±1.130	-.607 [2.559;-3.773]	p=.704
	Δ4	3.631±1.264	5.382±1.204	-1.751 [1.726;-5.228]	p=.319
**PSG Arousal** (events)	Δ1	-3.998±.765	-3.890±.773	-.109 [2.050;-2.1267]	p=.921
	Δ4	-5.750±1.146	-5.640±1.078	-.110 [3.026;-3.247]	p=.944

† Controlled for baseline levels of pain intensity (i.e., Brief Pain Inventory Average Pain Intensity)
‡ Controlled for baseline levels of insomnia severity (i.e., Insomnia Severity Index)

Data are Estimated Marginal (EM) Means ± Standard Error (SE) unless otherwise indicated. Includes all primary and secondary outcomes. Higher scores represent worse outcomes, except for Pressure Pain Thresholds, Polysomnography-derived sleep quality, SF36 mental and physical health and physical activity percentages.

CBTi-BEPM: Cognitive Behavioral Therapy for Insomnia integrated in Best-Evidence Pain Management; BEPM: Best-Evidence Pain Management; PSG: Polysomnography

Δ1= Baseline – timepoint 1 (immediate post-intervention)
Δ2= Baseline – timepoint 2 (3 months post-intervention)
Δ3= Baseline – timepoint 3 (6 months post-intervention)
Δ4= Baseline – timepoint 4 (12 months post-intervention, primary endpoint)

Table 8. First sensitivity analyses, controlling for baseline levels of pain intensity or insomnia severity for respectively the pain-related and sleep-related outcomes.

© 2024 Malfliet A et al. *JAMA Network Open.*

Two Forest Plots are presented to visually show the results of the first sensitivity analysis at post-intervention and 1 year follow-up, with regard to self-reported pain outcomes (primary outcome and secondary, exploratory pain-outcomes - eFigure 4) and secondary, exploratory self-reported sleep outcomes (eFigure 5).

eFigure 3. Forest Plot showing the results of the first sensitivity analyses using delta values for self-reported pain-related outcomes.

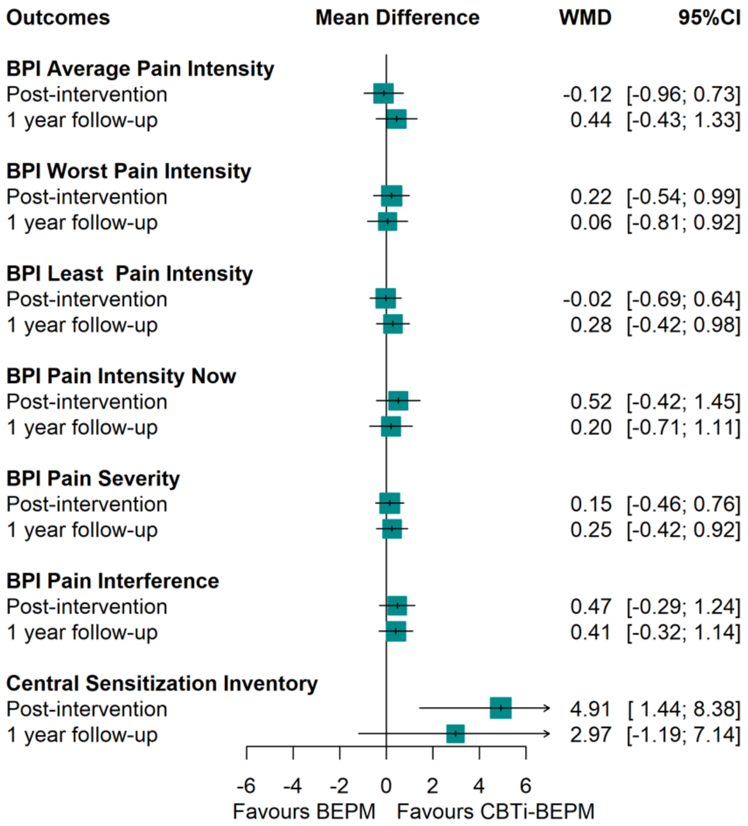

Legend. BPI: Brief Pain Inventory; WMD: Weighted Mean Differences; CBTi-BEPM: Cognitive Behavioral Therapy for Insomnia integrated in Best-Evidence Pain Management; BEPM: Best-Evidence Pain Management

© 2024 Malfliet A et al. *JAMA Network Open.*

eFigure 4. Forest Plot showing the results of the first sensitivity analyses using delta values for self-reported sleep-related outcomes.

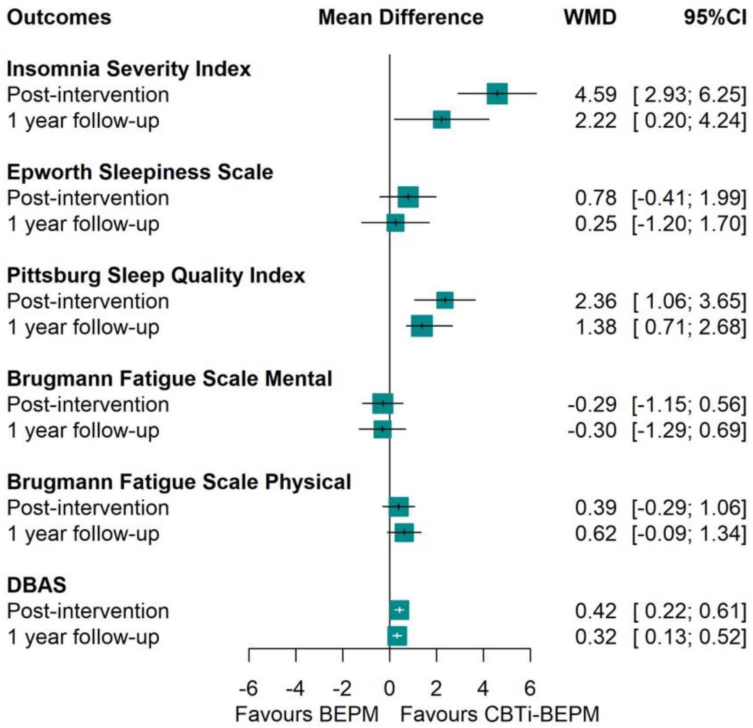

Legend. DBAS: Dysfunctional Beliefs About Sleep; WMD: Weighted Mean Differences; CBTi-BEPM: Cognitive Behavioral Therapy for Insomnia integrated in Best-Evidence Pain Management; BEPM: Best-Evidence Pain Management

© 2024 Malfliet A et al. *JAMA Network Open.*

eTable 5. Second sensitivity analyses, comparing dropouts to non-dropouts

	Dropout (incl. loss-to-follow-up) N = 22	Retainer / non-dropout N = 101	Mean group difference [95% CI]	p-value
Pain duration (months)[a]	72.88 (65.75)	93.73 (101.55)	-20.860 [-64.028;22.309]	p=.095
Age[a]	35.67 (7.89)	41.49 (11.44)	-5.828 [-10.717;-.939]	p=.015
Group allocation[b]	E=10 ; C=14	E=52 ; C=47	n.a.	p=.340
Sex[b]	M=10 ; F=14	M=31; F=68	n.a.	p=.334
Pain location[b]	N=17 ; B=7	N=53 ; B=46	n.a.	p=.125
Education[b]	13/5/5/1	37/42/20/0	n.a.	p=.050
Body Mass Index[a]	23.45 (2.75)	23.30 (3.24)	.148 [-1.272;1.568]	p=.273
BPI Average[a]	5.04 (1.94)	4.52 (2.06)	.527 [-.391;1.444]	p=.496
BPI Interference[a]	3.65 (1.85)	3.04 (1.83)	.607 [-.221;1.434]	p=.772
CSI[a]	47.25 (11.86)	42.87 (2.67)	4.381 [-.453;9.215]	p=.530
PSQI[a]	9.75 (2.67)	9.47 (2.70)	.285 [-.929;1.500]	p=.675
ISI[a]	15.92 (4.96)	14.95 (3.91)	.967 [-.894;2.829]	p=.213
HADS Anxiety[a]	9.75 (3.77)	8.53 (3.56)	1.225 [-.396;2.846]	p=.608
HADS Depression[a]	5.17 (3.74)	5.16 (3.21)	.005 [-1.490;1.500]	p=.286

Abbreviations:
BPI = Brief Pain Inventory; CSI = Central Sensitization Inventory; PSQI = Pittsburgh Sleep Quality Index; ISI = Insomnia Severity Index; HADS = Hospital Anxiety and Depression Scale; E = Experimental; C = Control; M Male; F = Female; N = Neck pain; B = Back pain; n.a. = not applicable.

[a] Continuous variable, presented as mean (SD). Group differences checked using independent samples t-test.
[b] Categorical variable, presented as frequencies. Group differences checked using Chi-Square test.

.

© 2024 Malfliet A et al. *JAMA Network Open.*

eTable 6. Remitters, responders and NNT analyses.

	EXPERIMENTAL CBTi + BEPM		CONTROL BEPM-only		NUMBERS NEEDED TO TREAT	
	Directly post treatment	12 months follow-up	Directly post treatment	12 months follow-up	Directly post treatment [95%CI]	12 months follow-up [95%CI]
**Responder rates BPI** [a]	19/54 (35.2%)	20/47 (42.6%)	15/54 (27.8%)	10/52 (19.2%)	14 [7-19]	4 [2-6]
**Remission rates ISI** [b]	49/54 (90.1%)	41/47 (87.2%)	38/54 (70.4%)	41/52 (78.8%)	5 [4-6]	12 [8-16]
**Responder rates ISI** [c]	43/54 (79.6%)	30/47 (63.8%)	20/54 (37.0%)	20/52 (38.5%)	2 [1-3]	4 [3-5]

Abbreviations:
BPI = Brief Pain Inventory; ISI = Insomnie Severity Index; CBTi = Cognitive Behavioral Therapy for Insomnia; BEPM = Best Evidence Physical Therapy

[a] surpassed the BPI MCID of 30%
[b] scores below or equal to 14
[c] surpassed the ISI MCID of 6 points

© 2024 Malfliet A et al. *JAMA Network Open.*

eTable 7. Success of assessor and participant blinding

	Unsure (score = 50)	Rated incorrect	Rated correct
Assessor blinding			
T1 (n rated = 106)	95% (n = 101)	0% (n = 0)	5% (n = 5)
T4 (n rated = 77)	88.3% (n = 68)	0% (n = 0)	11.7% (n = 9)
Participant blinding			
T4 (n rated = 55)	49.1% (n = 27)	23.6% (n = 13)	27.3% (n = 15)

Assessment of success of blinding procedure: The assessor was asked to indicate their idea of patient allocation at the assessment directly after post-treatment and at 12-month follow-up. A numeric rating scale (NRS; 0-100) was used, with 0 indicating '100% sure of participation in control group' and 100 indicating '100% sure of participation in experimental group'. A score of 50 meant that they had no idea about treatment allocation. The same was asked of the participants, but only after the 12-months follow-up assessment.

T1 = assessment directly post-treatment; T4 = assessment at 12 months follow-up

© 2024 Malfliet A et al. *JAMA Network Open.*

eTable 8. Absolute values at each timepoint.

		Experimental treatment		Control treatment		Mean group difference [95% CI]	Effect size
		EM Means±SE	% change relative to baseline	EM Means±SE	% change relative to baseline		
**Brief Pain Inventory Average Pain Intensity** (0-10)	T0	4.984±.256	-	4.246±.258	-	-.738 [-1.457;-.019]	-
	T1	3.373±.313	-32.32	2.841±.318	-33.09	-.532 [-1.416;.352]	.215
	T2	3.588±.312	-28.01	3.145±.318	-25.93	-.443 [-1.325;.440]	.179
	T3	3.232±.291	-35.15	2.878±.291	-32.22	-.354 [-1.171;.462]	.155
	T4	2.975±.316	-40.31	3.244±.312	-23.60	.269 [-.612;1.149]	.109
**Brief Pain Inventory Worst** (0-10)	T0	7.161±.202	-	6.934±.204	-	-.227 [-.795;.341]	-
	T1	5.948±.281	-16.94	5.994±.285	-13.56	.046 [-.747;.839]	.021
	T2	6.237±.302	-19.17	5.788±.307	-10.05	-.449 [-1.302;.403]	.188
	T3	5.479±.314	-23.49	5.806±.314	-10.05	.057 [-.823;.938]	.133
	T4	6.035±.321	-15.72	5.948±.316	-14.22	-.087 [-.979;.804]	.035
**Brief Pain Inventory Least** (0-10)	T0	2.419±.207	-	1.934±.209	-	-.485 [-1.068;.098]	-
	T1	1.555±.174	-35.72	1.243±.177	-35.73	-.312 [-.804;.181]	.227
	T2	1.461±.183	-39.60	.897±.186	-53.62	-.564 [-1.080;-.048]	.390
	T3	1.465±.216	-39.44	1.201±.216	-37.90	-.264 [-.869;.340]	.156
	T4	1.072±.186	-55.68	1.043±.183	-46.07	-.028 [-.546;.489]	.020
**Brief Pain Inventory Now** (0-10)	T0	4.274±.270	-	3.230±.272	-	-1.045 [-1.802;-.287]	-
	T1	2.619±.330	-38.72	2.423±.335	-24.98	-.196 [-1.128;.736]	.075
	T2	3.037±.311	-28.94	2.057±.316	-36.32	-.980 [-1.859;-.101]	.399
	T3	2.658±.309	-37.81	2.362±.309	-26.87	-.295 [-1.162;.571]	.122
	T4	2.598±.318	-39.21	2.363±.312	-26.84	-.236 [-1.119;.348]	.095
**Brief Pain Inventory Severity** (0-10)	T0	4.710±.192	-	4.086±.193	-	-.624 [-1.163;-.085]	
	T1	3.371±.227	-28.43	3.118±.230	-23.69	-.252 [-.893;.388]	.141
	T2	3.580±.238	-23.99	2.965±.242	-27.43	-.615 [-1.288;.058]	.327
	T3	3.278±.234	-30.40	3.054±.235	-25.25	-.224 [-.883;.434]	.122
	T4	3.164±.237	-32.82	3.151±.234	-22.88	-.013 [-.637;.647]	.007
**Brief Pain Inventory Interference** (0-10)	T0	3.551±.230	-	2.763±.232	-	-.787 [-1.433;-.141]	-
	T1	1.909±.254	-46.24	1.830±.258	-33.77	-.078 [-.796;.640]	.039
	T2	1.759±.254	-66.69	1.183±.259	-36.33	.054 [-.665;.773]	.286
	T3	1.777±.260	-49.96	1.746±.260	-36.81	-.030 [-.758;.697]	.015
	T4	1.752±.250	-50.66	1.678±.248	-39.27	-.074 [-.772;.625]	.038

© 2024 Malfliet A et al. *JAMA Network Open.*

**Central Sensitization Inventory** (0-100)	T0	44.526±1.374	-	43.048±1.386	-	-1.478 [-5.341;2.386]	-
	T1	31.915±1.550	-28.32	35.736±1.575	-16.99	3.821 [-.556;8.198]	.312
	T2	34.197±1.739	-23.20	34.296±1.767	-20.33	.099 [-4.812;5.010]	.007
	T3	33.720±1.842	-24.27	37.078±1.856	-13.87	3.357 [-1.823;8.537]	.232
	T4	32.587±1.752	-26.81	35.792±1.752	-16.85	2.205 [-2.705;2.705]	.495
**Pressure Pain Thresholds Primary*** (kgf)	T0	4.856±.295	-	4.902±.292	-	.046 [-.776;.868]	-
	T1	6.088±.347	+25.3	5.596±.348	+14.16	-.492 [-1.465;.481]	.181
	T4	6.009±.400	+23.74	6.128±.386	+25.01	.120 [-.982;1.222]	.039
**Pressure Pain Thresholds Calf Secondary** (kgf)	T0	5.196±.239	-	4.729±.237		-.467 [-1.132;.198]	-
	T1	5.700±.269	+9.70	5.107±.269	+7.99	-.593 [-1.346;.160]	.281
	T4	6.020±.312	+15.86	5.560±.303	+17.57	-.460 [-1.323;.401]	.191
**Pressure Pain Thresholds Hand Secondary** (kgf)	T0	4.171±.203	-	4.065±.201	-	-.106 [-.673;.460]	-
	T1	4.755±.221	+14.00	4.248±.221	+4.50	-.506 [-1.126;.113]	.293
	T4	5.034±.264	+20.70	4.768±.255	+17.29	-.266 [-.994;.462]	.131
**Insomnia Severity Index** (0-28)	T0	16.000±.515	-	14.262±.519	-	-1.738 [-3.186;-.289]	-
	T1	7.908±.623	-50.57	11.499±.633	-19.37	3.592 [1.831;5.352]	.729
	T2	8.094±.666	-49.41	10.442±.678	-26.78	2.348 [.465;4.231]	.445
	T3	8.038±.724	-49.76	10.745±.726	-26.78	2.707 [.674;4.740]	.476
	T4	8.701±.750	-45.62	10.154±.744	-28.80	1.454 [-.640;3.547]	.248
**Epworth Sleepiness Scale** (0-24)	T0	8.258±.593	-	8.213±.597	-	-.045 [-1.711;1.621]	-
	T1	7.108±.541	-13.92	7.839±.548	-4.55	.732 [-.795;2.258]	.171
	T2	6.791±.586	-17.76	7.182±.594	-12.55	.391 [-1.262;2.044]	.084
	T3	6.509±.575	-21.18	7.128±.578	-13.21	.619 [-.997;2.234]	.137
	T4	6.792±.556	-17.75	6.937±.554	-17.30	.146 [-1.149;1.701]	.033
**Pittsburg Sleep Quality Index** (0-21)	T0	9.839±.340	-	9.197±.343	-	-.642 [-1.598;.314]	-
	T1	5.343±.368	-45.70	7.127±.375	-22.51	1.785 [.742;2.827]	.612
	T2	5.400±.364	-45.12	6.416±.370	-30.24	1.015 [-.013;2.044]	.353
	T3	5.654±.402	-42.53	6.764±.401	-26.45	1.110 [-.016;2.237]	.353
	T4	5.880±.410	-40.24	6.758±.405	-26.52	.878 [-.264;2.021]	.275
**Brugmann Fatigue Scale Mental** (0-12)	T0	3.129±.318	-	3.328±.321	-	.199 [-.696;1.094]	-
	T1	2.193±.253	-29.91	2.389±.257	-28.21	.196 [-.518;.910]	.098
	T2	1.973±.273	-36.94	2.107±.277	-36.69	.134 [-.637;.904]	.062
	T3	1.876±.295	-40.04	2.430±.295	-26.98	.554 [-.274;1.381]	.239
	T4	2.133±.287	-31.83	2.297±.285	-30.98	.163 [-.639;.965]	.073
	T0	3.565±.275	-	3.180±.277	-	-.384 [-1.157;.388]	-

© 2024 Malfliet A et al. *JAMA Network Open.*

**Brugmann Fatigue Scale Physical** (0-12)	T1	2.610±.237	-26.79	2.930±.241	-7.86	.320 [-.350;.991]	.171
	T2	2.409±.261	-32.43	2.814±.265	-7.86	.405 [-.333;1.143]	.196
	T3	2.282±.253	-35.99	3.097±.253	-2.61	.815 [.106;1.523]	.411
	T4	2.242±.248	-37.11	2.619±.245	-17.64	.376 [-.314;1.067]	.195
**DBAS** (0-10)	T0	2.977±.075	-	2.998±.076	-	.001 [-.210;.212]	-
	T1	2.297±.076	-22.84	2.730±.077	-8.94	.433 [.218;.648]	.722
	T2	2.253±.081	-24.32	2.676±.082	-10.74	.423 [.195;.651]	.662
	T3	2.204±.083	-25.97	2.630±.083	-12.27	.472 [.193;.660]	.654
	T4	2.249±.081	-24.45	2.589±.081	-13.64	.340 [.113;.567]	.535
**PSG – Sleep Onset Latency** (min)	T0	13.561±2.281	-	16.133±2.281	-	2.572 [-3.814;8.958]	-
	T1	11.810±1.303	-12.91	11.510±1.348	-28.65	-.300 [-4.025;3.424]	.029
	T4	11.273±3.202	-16.87	17.784±2.977	+10.23	6.511 [-2.195;15.217]	.269
**PSG Wake After Sleep Onset** (min)	T0	37.267±4.036	-	43.166±4.027	-	5.900 [-5.394;17.193]	-
	T1	29.981±4.612	-19.55	27.915±4.684	-35.33	-2.067 [-15.115;10.982]	.057
	T4	40.652±7.543	9.08	35.051±6.727	-18.80	-5.600 [-25.755;14.554]	.100
**PSG Early Morning Awakenings** (min)	T0	5.013±1.085	-	5.769±1.085	-	.756 [-2.281;3.793]	-
	T1	6.038±1.754	20.45	9.120±1.803	58.09	3.083 [-1.907;8.072]	.221
	T4	7.887±3.441	57.33	14.099±3.191	144.39	6.212 [-3.135;15.559]	.239
**PSG Time In Bed** (min)	T0	476.097±9.789	-	492.758±9.767	-	16.661 [-10.732;44.055]	-
	T1	463.404±10.266	-2.67	480.756±10.478	-2.43	17.353 [-11.768;46.473]	.213
	T4	487.786±10.944	2.45	496.023±10.039	0.66	8.237 [-21.331;37.805]	.100
**PSG Total Sleep Time** (min)	T0	424.535±8.149	-	433.296±8.131	-	8.761 [-14.043;31.565]	-
	T1	413.646±10.165	-2.56	432.100±10.295	-0.28	18.454 [-10.260;47.168]	.230
	T4	424.586±11.490	0.01	427.825±10.438	-1.26	3.239 [-27.683;34.161]	.038
**PSG Sleep Efficiency** (%)	T0	89.943±.848	-	88.875±.848	-	-1.067 [-3.442;1.308]	-
	T1	89.504±1.053	-0.49	89.781±1.080	1.01	.277 [-2.715;3.269]	.033
	T4	87.863±1.649	-2.31	86.448±1.543	-2.73	-1.416 [-5.199;3.080]	.113
**PSG REM-sleep** (%)	T0	16.051±.874	-	14.231±.874	-	-1.820 [-4.266;.627]	-
	T1	20.512±.851	27.79	19.021±.873	33.66	-1.492 [-3.910;.926]	.220
	T4	19.986±1.093	24.51	19.290±1.024	35.55	-.695 [-3.674;2.284]	.084
**PSG non-REM-sleep** (%)	T0	83.944±.956	-	86.289±.956	-	2.344 [-.331;5.020]	-
	T1	79.488±.857	-5.31	81.118±.879	-5.99	1.630 [-.804;4.064]	.239
	T4	80.017±1.103	-4.68	80.915±1.034	-6.23	.903 [-2.104;3.910]	.107
**PSG Arousal** (events)	T0	4.618±.289	-	4.515±.289	-	-.103 [-1.912;.705]	-
	T1	8.458±.717	83.15	8.407±.737	86.20	-.051 [-2.092;1.989]	.009

© 2024 Malfliet A et al. *JAMA Network Open.*

	T4	10.497±1.196	127.31	9.756±1.115	116.08	-.741 [-3.997;2.515]	.082
**SF36 Mental** (0-400)	T0	248.471±9.940	-	257.892±9.787	-	9.421 [-18.210;37.052]	-
	T1	294.571±9.455	18.55	291.881±9.364	13.18	-2.690 [-29.082;23.702]	.036
	T2	299.986±10.134	20.73	292.869±10.038	13.56	7.117 [-35.408;21.174]	.090
	T3	295.398±10.168	18.89	279.455±9.902	8.36	-15.942 [-44.087;12.202]	.203
	T4	287.780±11.208	15.82	293.073±10.821	13.64	5.283 [-25.623;36.190]	.061
**SF36 Physical** (0-400)	T0	213.351±8.724	-	242.257±8.590	-	28.905 [4.655;53.156]	-
	T1	294.568±9.082	38.07	298.834±8.999	23.35	4.266 [-21.092;29.623]	.060
	T2	287.745±10.634	34.87	294.429±10.537	21.54	6.684 [-36.376;23.008]	.081
	T3	284.613±10.630	33.40	281.999±10.342	16.40	-2.614 [-32.031;26.803]	.032
	T4	289.524±10.371	36.56	291.385±10.058	19.54	1.861 [-30.521;26.799]	.023
**Hospital Anxiety and Depression Scale Anxiety** (0-21)	T0	8.581±.461	-	8.951±.464	-	.370 [-.925;1.665]	-
	T1	6.113±.475	-28.76	7.190±.482	-19.67	1.077 [-.264;2.417]	.287
	T2	6.291±.474	-26.69	6.953±.481	-22.32	.662 [-.676;1.999]	.177
	T3	6.031±.480	-29.72	4.760±.481	-46.82	1.729 [.382;3.075]	.337
	T4	5.953±.459	-30.62	7.252±.456	-18.98	1.299 [.016;2.582]	.362
**Hospital Anxiety and Depression Scale Depression** (0-21)	T0	5.290±.421	-	5.033±.425	-	-.258 [-1.442;.926]	-
	T1	3.069±.412	-41.98	3.888±.418	-22.75	.819 [-.343;1.981]	.252
	T2	3.140±.440	-40.64	4.058±.447	-19.37	.918 [-.325;2.161]	.264
	T3	3.316±.431	-37.32	4.341±.434	-13.75	1.025 [-.187;2.238]	.302
	T4	2.995±.433	-43.38	3.754±.431	-25.41	0.759 [-.452;1.969]	.224
**Physical Activity Sedentary** (%)	T0	49.215±.912	-	48.432±.917	-	-.783 [-3.345;1.799]	-
	T1	49.722±.976	1.03	48.230±.989	-0.42	-1.492 [-4.247;1.262]	.194
	T4	49.575±1.055	0.73	48.477±1.045	0.09	-1.098 [-4.051;1.855]	.133
**Physical Activity Light** (%)	T0	38.994±.754	-	39.648±.759	-	.654 [-1.464;2.772]	-
	T1	38.399±.808	-1.53	39.381±.821	-0.67	.982 [-1.302;3.266]	.154
	T4	38.943±.867	-0.13	39.556±.859	-0.23	.613 [-1.814;3.040]	.091
**Physical Activity Moderate** (%)	T0	11.792±.532	-	11.926±.534	-	.133 [-1.358;1.625]	-
	T1	11.865±.617	0.62	12.527±.618	5.04	.662 [-1.068;2.393]	.137
	T4	11.537±.594	-2.16	11.717±.590	-1.75	.180 [-1.481;1.841]	.039
**Step Count**	T0	13153.525±398.681	-	13600.396±400.345	-	446.872 [-671.775;1565.519]	-
	T1	13106.462±434.756	-0.36	13819.283±436.867	1.60	712.821 [-508.750;1934.392]	.209
	T4	13233.600±416.183	0.61	13198.525±412.607	-2.95	-35.075 [-1197.562;1127.411]	.011
Cohen's D is interpreted as 'very large' (>1.3), 'large' (.80-1.29), 'medium' (.50-.79), 'small' (.20-.49), 'negligible' (<.20).							

Table 12. Absolute values at each timepoint.

© 2024 Malfliet A et al. *JAMA Network Open.*

eReferences

1. Nilius G, Domanski U, Schroeder M, et al. A randomized controlled trial to validate the Alice PDX ambulatory device. *Nature and science of sleep.* 2017;9:171-180.
2. Berry RB, Brooks R, Gamaldo CE, Harding SM, Marcus C, Vaughn BV. The AASM manual for the scoring of sleep and associated events. *Rules, Terminology and Technical Specifications, Darien, Illinois, American Academy of Sleep Medicine.* 2012;176:2012.
3. Wall P, Melzack R. Textbook of Pain. 1999.
4. van Wilgen CP, Nijs J. Pijneducatie - een praktische handleiding voor (para)medici. 2010.
5. Butler DS, Moseley GL. Explain Pain. 2003.
6. Nijs J, Meeus M, Cagnie B, et al. A modern neuroscience approach to chronic spinal pain: combining pain neuroscience education with cognition-targeted motor control training. *Physical therapy.* 2014;94:730-738.
7. Malfliet A, Kregel J, Meeus M, et al. Applying contemporary neuroscience in exercise interventions for chronic spinal pain: treatment protocol. *Brazilian journal of physical therapy.* 2017;21.
8. Nijs JJ, Lluch Girbes E, Lundberg M, et al. Exercise therapy for chronic musculoskeletal pain: Innovation by altering pain memories. *Manual therapy.* 2015;20:216-220.
9. Van Looveren E, Meeus M, Cagnie B, et al. Combining Cognitive Behavioral Therapy for Insomnia and Chronic Spinal Pain Within Physical Therapy: A Practical Guide for the Implementation of an Integrated Approach. *Physical Therapy.* 2022;102(8).

© 2024 Malfliet A et al. *JAMA Network Open.*
