# Supplementary material for: Cognitive Behavioral Therapy for Insomnia in Pain Management for Nonspecific Chronic Spinal Pain: A Randomized Clinical Trial
Source: JAMA Netw Open. 2024 Aug 9;7(8):e2425856. doi: 10.1001/jamanetworkopen.2024.25856 (PMC11316234; doi:10.1001/jamanetworkopen.2024.25856)
Supplement: Supplement 3. — Data Sharing Statement [file jamanetwopen-e2425856-s003.pdf]

Data Sharing Statement

Malfliet. Cognitive Behavioral Therapy for Insomnia in Pain Management for Nonspecific Chronic Spinal Pain. *JAMA Netw Open*. Published August 09, 2024.
doi:10.1001/jamanetworkopen.2024.25856

Data
Data available: No

Additional Information
Explanation for why data not available: Because of the highly personal and sensitive nature of the data, this will not be made publicly available. However, any request to share data will be considered by the consortium, and if needed, a data sharing agreement will be installed prior to any sharing of data.
